# Beyond Body Weight: Maternal Propolis Selectively Modulates Metabolic and Hepatic Outcomes in Male and Female Rat Offspring Exposed to a Maternal Cafeteria Diet

**DOI:** 10.3390/nu18172881

**Published:** 2026-09-02

**Authors:** Kadriye Elif İmre, Aslı Akyol

**Affiliations:** 1 Department of Nutrition and Dietetics, Kastamonu University, Kastamonu 37150, Turkey; keimre@kastamonu.edu.tr; 2 Department of Nutrition and Dietetics, Hacettepe University, Ankara 06100, Turkey

**Keywords:** propolis, developmental origins of health and disease, cafeteria diet, obesity, metabolism

## Abstract

**Background/Objectives**: Maternal obesogenic diets may produce persistent developmental alterations in offspring, with response patterns that may differ between males and females. We investigated whether propolis supplementation from preconception through lactation modified metabolic and hepatic outcomes in offspring exposed to a maternal cafeteria diet. **Methods**: Thirty-two Wistar rat dams received a control diet (CON), cafeteria diet (CAF), or either diet with propolis in drinking water (CONP and CAFP; target exposure, 200 mg/kg/day). One male and one female per litter (*n* = 8/sex/group) received standard chow after weaning. This design isolated maternal exposure because offspring received no propolis after weaning. At 18 weeks, metabolic and inflammatory biomarkers, liver histopathology, and hepatic lipid-metabolism gene expression were evaluated using groupwise tests and two-way ANOVA. **Results**: Final body weights were comparable across groups. In males, maternal diet affected glucose (*p* = 0.034) and HOMA-IR (*p* = 0.023), with a diet × propolis interaction for glucose (*p* = 0.042). CAF males had higher TNF-α than CAFP and CONP males. In females, diet × propolis interactions were detected for insulin, total cholesterol, triglycerides, and leptin (all *p* ≤ 0.049); CAF females had higher triglycerides than CON and CAFP females. Hydropic degeneration was greater in CAF dams than all other groups and in CAF than CONP male offspring; female histopathology did not differ. Male hepatic gene expression did not differ among groups, whereas female PPAR-β and FADS2 showed propolis main effects (*p* = 0.046 and *p* = 0.009, respectively). **Conclusions**: Maternal cafeteria-diet exposure was associated with persistent metabolic and hepatic alterations despite comparable offspring body weights, with different response patterns observed in the separate male and female analyses. Maternal propolis supplementation selectively modified some inflammatory, lipid-related, and histopathological outcomes; however, these effects were heterogeneous and did not consistently normalize the cafeteria-associated phenotype. Overall, the findings support partial, endpoint-specific, and context-dependent modulation rather than uniform metabolic or hepatic protection.

## 1. Introduction

Obesity is among the most urgent global health challenges. Current projections indicate that by 2050 nearly 60% of adults—approximately 3.8 billion individuals—will be living with overweight or obesity [1]. This burden increasingly affects women entering pregnancy. A recent global systematic review estimated the prevalence of maternal obesity at 20.9% and projected an increase to 23.3% by 2030 [2]. Beyond its immediate obstetric consequences, maternal obesity is increasingly recognized as a determinant of long-term metabolic vulnerability in offspring. Experimental obesogenic dietary exposure provides an important means of investigating the developmental processes underlying this association.

Experimental evidence indicates that maternal exposure to high-fat or highly palatable diets can induce persistent alterations in offspring adiposity, dietary preference, endocrine function, and metabolic regulation, with differing response patterns reported in male and female offspring [3,4]. Maternal nutritional and metabolic disturbances may affect placental nutrient transport and intrauterine nutrient signaling, thereby influencing fetal growth and developmental trajectories [5]. Associations between maternal and neonatal one-carbon metabolite profiles and infant DNA methylation patterns further support the involvement of nutrition-sensitive epigenetic processes [6]. Evidence from animal models similarly indicates that maternal obesity and obesogenic dietary exposure can program multiple metabolic tissues and increase offspring susceptibility to obesity, insulin resistance, and related metabolic disorders later in life [7].

The Developmental Origins of Health and Disease (DOHaD) framework provides a conceptual basis for understanding how early-life exposures influence later disease susceptibility [8]. According to this framework, nutritional and metabolic disturbances during the periconceptional, gestational, and early postnatal periods may induce persistent adaptations in organ development and metabolic regulation [8,9]. Epigenetic mechanisms, including DNA methylation, histone modifications, and non-coding RNA-mediated regulation, are considered important mediators linking the maternal nutritional environment to long-term offspring phenotypes [10].

Dietary patterns constitute a major modifiable component of the maternal environment. Controlled human evidence has shown that an ad libitum ultra-processed diet increases energy intake and body weight compared with an unprocessed diet [11]. In experimental research, high-fat diets are widely used to model obesity and insulin resistance, although their metabolic effects vary according to dietary composition and fatty acid profile [12]. Cafeteria diet models expose rodents to a variety of highly palatable, energy-dense foods and can reproduce several metabolic disturbances associated with contemporary obesogenic dietary environments [13,14]. Maternal cafeteria diet exposure can produce lasting alterations in offspring adiposity, hepatic metabolism, and inflammatory regulation, with heterogeneous response patterns reported across studies of male and female offspring [15,16,17]. The liver is particularly relevant in this context because it plays a central role in systemic glucose and lipid homeostasis and is sensitive to nutritional disturbances occurring during early development [18].

Given the increasing prevalence of maternal obesity and obesogenic dietary exposure, identifying nutritional strategies that may mitigate adverse developmental programming has become an important research priority. Propolis is a resinous bee product containing diverse bioactive constituents, including polyphenols, flavonoids, terpenoids, and phenolic acids [19]. Preclinical evidence suggests that propolis and its bioactive compounds may exert antioxidant, anti-inflammatory, glucose-regulatory, and lipid-modulatory effects [20]. However, most available evidence derives from the direct administration of propolis in adult disease models. It remains unclear whether propolis administered to dams before and during pregnancy and lactation can modify metabolic and hepatic programming in offspring after the maternal intervention has ceased.

Accordingly, the present study investigated the long-term effects of maternal cafeteria diet exposure and whether concurrent maternal propolis supplementation modified these effects in adult rat offspring. Dams received standard chow or a cafeteria diet, with or without propolis administered in drinking water, during preconception, gestation, and lactation, whereas all offspring received standard chow without propolis after weaning. Maternal dietary intake and body and organ weights were evaluated together with sex-stratified offspring metabolic and inflammatory biomarkers, liver histopathology, and hepatic mRNA expression of selected genes involved in lipid metabolism, including *Cyp7a1*, *Ppara*, *Ppard*, *Srebf1*, and *Fads2*. We hypothesized that maternal cafeteria diet exposure would induce metabolic and hepatic alterations in offspring and that maternal propolis supplementation would modify these effects; responses were evaluated separately in male and female offspring.

## 2. Materials and Methods

### 2.1. Phytochemical Characterization and Selection of the Propolis Preparation

To identify a water-based propolis preparation suitable for administration through drinking water, five water-based and five ethanol-based commercial propolis formulations obtained from Turkish manufacturers were screened. Total phenolic content (TPC) and total flavonoid content (TFC) were determined in duplicate at the Central Research Laboratory of Çankırı University using a DR 6000 UV–Vis spectrophotometer (Hach Lange, Düsseldorf, Germany).

TPC was determined in duplicate using the Folin–Ciocalteu colorimetric method [21,22]. Propolis samples were dissolved in 95% ethanol at a concentration of 50 mg/mL and diluted as appropriate. The samples were mixed with 10-fold-diluted Folin–Ciocalteu reagent, followed by the addition of saturated sodium carbonate solution to establish alkaline conditions (approximately pH 10). After incubation at room temperature for 2 h, absorbance was measured at 765 nm using a DR 6000 UV–Vis spectrophotometer (Hach Lange, Düsseldorf, Germany). TPC was calculated using a gallic acid calibration curve and expressed as milligrams of gallic acid equivalents per gram of extract (mg GAE/g extract).

TFC was determined in duplicate using the aluminum nitrate colorimetric method [23]. Propolis samples were diluted with 80% aqueous ethanol and added to a reaction mixture containing 10% aluminum nitrate, 1 M potassium acetate, and 80% ethanol. Following incubation at room temperature for 40 min, absorbance was measured at 415 nm using the same UV–Vis spectrophotometer. TFC was calculated using a quercetin calibration curve and expressed as milligrams of quercetin equivalents per gram of extract (mg QE/g extract).

Because propolis was administered through drinking water, only water-based preparations were considered suitable for the animal intervention; ethanol-based formulations were included solely for comparative phytochemical characterization. The commercially available water-based preparation with the highest TPC and TFC values was selected. Detailed phytochemical screening results are presented in Supplementary Table S1.

### 2.2. Animals and Experimental Design

All experimental procedures were conducted at the Kastamonu University Animal Research and Application Unit and were approved by the Kastamonu University Local Ethics Committee for Animal Experiments (approval no. 2023-31). The study was reported in accordance with the ARRIVE 2.0 guidelines [24], and the completed ARRIVE checklist is provided as Supplementary File S1.

Thirty-two 4-week-old virgin female Wistar rats were obtained from the Karabük University Experimental Medicine Research Center. Animals were housed individually in polycarbonate cages under controlled environmental conditions (22–24 °C; 12 h light/12 h dark cycle) with ad libitum access to food and drinking water. Following a 7-day acclimatization period, the dams were randomly allocated to four experimental groups using a random-number method:

1. Standard chow without propolis supplementation (CON; *n* = 8);
2. Standard chow with propolis provided through drinking water (CONP; *n* = 8);
3. Cafeteria diet without propolis supplementation (CAF; *n* = 8); and
4. Cafeteria diet with propolis provided through drinking water (CAFP; *n* = 8).

The preconception intervention lasted 8 weeks. Thereafter, the assigned dietary and propolis interventions were continued without interruption throughout gestation and lactation until weaning. Accordingly, the 14-week maternal intervention period reported for dietary intake refers to the total exposure period spanning preconception, gestation, and lactation. The standard chow was a commercially available laboratory diet (catalog no. RT-01; DSA Tarım Ürünleri, Yahşihan/Kırıkkale, Türkiye). The diet provided an energy density of 2646.31 kcal/kg, with carbohydrates, protein, and fat contributing 69.65%, 20.67%, and 9.68% of total energy, respectively. On an as-fed basis, the diet contained 80.5 g/kg crude fiber, 72.1 g/kg ash, 42.2 g/kg sugars, and 299.5 g/kg starch. The detailed nutritional composition of the standard chow, including its mineral, vitamin, and trace nutrient contents, is presented in Supplementary Table S2.

The cafeteria-diet model consisted of standard chow offered alongside a rotating selection of highly palatable human snack foods rich in fat, sugar, and salt, based on previously described cafeteria-diet models [13,25]. Fifteen food items were used: milk chocolate, caramel chocolate, pistachio chocolate, peanut nougat chocolate, cream-filled cocoa biscuits, hazelnut biscuits, cocoa-filled biscuits, caramel-coated biscuits, fresh Kashar cheese, potato chips, cheese-flavored corn chips, cheese crackers, sandwich crackers, corn snacks, and salted peanuts. Five cafeteria-diet items were provided each day, and three items were replaced daily to maintain dietary novelty and variety. Food and water were available ad libitum.

The CONP and CAFP groups received a commercially available water-based propolis preparation through drinking water at a target exposure of 200 mg/kg/day. The commercial preparation contained 80 mg of propolis per mL. Body weight and drinking-water consumption were recorded daily for each dam. The target daily propolis amount was calculated according to body weight as: target propolis amount (mg/day) = 200 mg/kg × body weight (kg). The corresponding volume of commercial propolis preparation was calculated as: commercial preparation (mL/day) = target propolis amount (mg/day)/80 mg/mL. The calculated volume of propolis preparation was added to the drinking-water volume provided for the subsequent 24-h period, based on the amount of water consumed by the individual dam during the preceding 24 h. Body weight and water consumption were reassessed daily, and the volume of propolis preparation was recalculated and readjusted accordingly throughout the intervention. Because administration occurred ad libitum through drinking water, the timing and pattern of consumption within each 24-h period could not be controlled with the same precision as direct administration; therefore, 200 mg/kg/day is reported as the target body-weight-adjusted exposure rather than as an invariant delivered dose. The target exposure of 200 mg/kg/day was selected based on previous experimental work by Eraslan et al., in which propolis was administered to female rats at the same body-weight-adjusted exposure through drinking water and was associated with measurable changes in several serum biochemical parameters, including glucose, triglycerides, cholesterol, and liver-related enzymes [26]. Thus, the selected exposure level and administration route were based on an established preclinical exposure precedent. However, because the previous study was conducted in a non-pregnant toxicological model rather than a maternal developmental model, 200 mg/kg/day should be regarded as a previously used experimental target exposure rather than an established optimal exposure for maternal supplementation. The CON and CAF groups received drinking water without propolis. Standard chow and each cafeteria-diet item were weighed daily using a calibrated precision balance. Humane endpoints were predefined, and animals exhibiting severe distress, impaired mobility, or body-weight loss exceeding 20% would have been withdrawn from the study. No animals met these criteria, and no unexpected adverse events occurred.

### 2.3. Mating, Litter Management, and Offspring Follow-Up

At the end of the 8-week preconception intervention, the female rats were mated with proven breeder male Wistar rats. Pregnancy was confirmed by the presence of a vaginal plug. Dams continued receiving their assigned dietary exposure and propolis condition throughout gestation and lactation.

Litters were standardized to a maximum of eight pups whenever possible. At weaning, one male and one female offspring were randomly selected from each litter for follow-up, and the remaining pups were humanely euthanized. The litter was considered the experimental unit, and only one offspring of each sex from each litter contributed to the sex-stratified analyses, thereby avoiding litter-level pseudoreplication. The maternal group size of eight dams per experimental condition was consistent with litter-based sample sizes used in comparable maternal cafeteria-diet studies [16], while also reflecting the ethical principle of reducing unnecessary animal use within the approved experimental protocol. The study design and planned group allocation were approved by the institutional animal ethics committee.

**Figure 1:**
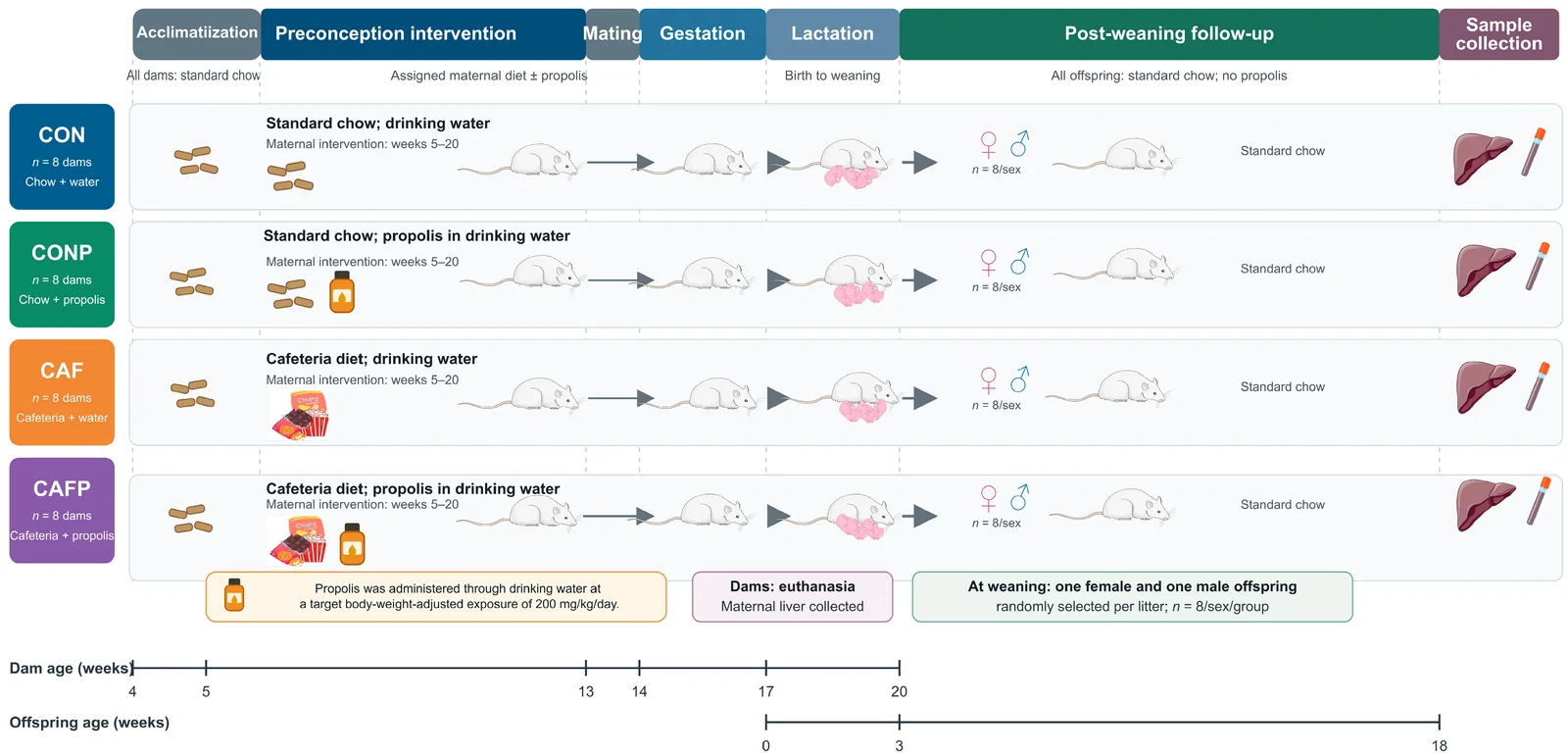
Experimental design and timeline of the maternal dietary intervention and offspring follow-up. Dams were assigned to the CON, CONP, CAF, and CAFP groups (*n* = 8/group), and the corresponding interventions were maintained throughout preconception, gestation, and lactation. Propolis was administered in drinking water at a target body-weight-adjusted exposure of 200 mg/kg/day. At weaning, one male and one female offspring were randomly selected from each litter (*n* = 8/sex/group) and maintained on standard chow without propolis until sample collection at 18 weeks of age. The schematic was conceptualized and designed by the authors using Inkscape (version 1.4.3). Abbreviations: CON, standard chow and drinking water; CONP, standard chow and propolis in drinking water; CAF, cafeteria diet and drinking water; CAFP, cafeteria diet and propolis in drinking water.

At the end of lactation, the dams were euthanized, and maternal liver samples were collected for histopathological evaluation. The selected offspring were maintained on standard chow without propolis supplementation from weaning until 18 weeks of age. At 18 weeks of age, the offspring were deeply anesthetized with ketamine (100 mg/kg) and xylazine (10 mg/kg). After confirmation of adequate anesthetic depth, blood was collected by cardiac puncture for biochemical analyses, euthanasia was completed by exsanguination, and liver samples were collected for histopathological and molecular analyses. The experimental design and timeline are presented in Figure 1.

Biochemical, molecular, and histopathological assessments were performed using coded samples by investigators blinded to group allocation.

### 2.4. Serum Biochemical and Inflammatory Analyses

Prior to terminal blood collection, all animals underwent a standardized 12-h overnight fast. Food was completely removed during this period, while plain drinking water remained available ad libitum. In the propolis-supplemented groups, propolis-containing drinking water was withdrawn at the start of the fasting period and replaced with clean, propolis-free drinking water. Blood samples were collected the following morning under deep anesthesia.

Serum insulin (E0707Ra), leptin (E0561Ra), adiponectin (E0758Ra), tumor necrosis factor-alpha (TNF-α; E0764Ra), and interleukin-10 (IL-10; E0108Ra) concentrations were measured using commercial rat-specific ELISA kits (Bioassay Technology Laboratory, Shanghai, China) according to the manufacturer’s instructions.

Serum glucose, triglyceride, and total cholesterol concentrations were determined using enzymatic colorimetric endpoint assays on a Mindray BS-400 automated biochemical analyzer (Mindray Medical, Shenzhen, China). Commercial kits specific for glucose (OttoBC142), triglycerides (OttoBC155), and total cholesterol (OttoBC135) were obtained from Otto Scientific (Ankara, Türkiye). All biochemical and ELISA analyses were conducted by Diagen Biotechnology Systems Inc. (Ankara, Türkiye) according to standardized laboratory procedures.

The homeostatic model assessment of insulin resistance (HOMA-IR) was calculated using the following equation [27,28]:

HOMA-IR = [fasting serum insulin (µU/mL) × fasting serum glucose (mg/dL)]/405.

### 2.5. Liver Histopathology

Liver samples from dams and offspring were fixed in 10% neutral-buffered formalin, processed using an automated tissue processor (HistoCore Pearl; Leica Biosystems, Nußloch, Germany), and embedded in paraffin. Sections of 5 µm thickness were prepared using a HistoCore MULTICUT microtome (Leica Biosystems), mounted on glass slides, deparaffinized in xylene, rehydrated through graded ethanol solutions, and stained with hematoxylin and eosin.

Slides were examined and photographed using a DM750 light microscope equipped with an ICC50 W digital camera (Leica Microsystems, Wetzlar, Germany). For each animal, two H&E-stained liver sections were evaluated. Across the sections from each animal, at least 10 randomly selected, non-overlapping microscopic fields were examined at ×200 magnification by an investigator blinded to the experimental groups. Hydropic degeneration, sinusoidal dilatation, and congestion were independently evaluated using a four-point semi-quantitative ordinal scale: 0, absent; 1, mild; 2, moderate; and 3, severe, adapted from the scoring approach described by Arslan et al. [29]. The severity category assigned to each field reflected the morphological severity of the corresponding histopathological feature. For each parameter, the individual animal-level score used for statistical analysis was calculated as the arithmetic mean of the field-level scores obtained from the two sections evaluated for that animal. Histological processing and evaluation were conducted at the Central Research Laboratory of Kastamonu University.

### 2.6. Hepatic Gene-Expression Analysis

Total RNA was isolated from offspring liver tissue using a commercial RNA extraction kit (TR-0877; Diagen Biotechnology Systems Inc., Ankara, Türkiye) according to the manufacturer’s instructions. RNA concentration and purity were evaluated spectrophotometrically, and complementary DNA was synthesized using a commercial reverse-transcription kit (Bioline Ltd., London, UK).

Quantitative real-time PCR was performed using the SensiFAST SYBR No-ROX Kit (Bioline Ltd., London, UK) on a CFX96 Real-Time PCR Detection System (Bio-Rad Laboratories, Hercules, CA, USA). The analyzed genes were cholesterol 7 alpha-hydroxylase (CYP7A1), peroxisome proliferator-activated receptor-alpha (PPAR-α), peroxisome proliferator-activated receptor-beta/delta (PPAR-β/δ), sterol regulatory element-binding protein-1c (SREBP-1c), and fatty acid desaturase 2 (FADS2). β-actin was used as the reference gene. Primer sequences are presented in Supplementary Table S3.

Relative mRNA expression was calculated using the 2^−ΔΔCt^ method after normalization to β-actin expression [30]. Gene-expression results are reported as relative fold changes. Before the correlation analyses, the derived fold-change variables were verified against the underlying gene-expression calculations. PPAR-α fold-change values were recalculated using the standard 2^−ΔΔCt^ transformation, and one transcription error in a female CAF FADS2 fold-change value was corrected following verification of the source calculation. All RNA isolation, reverse transcription, and qPCR procedures were conducted by Diagen Biotechnology Systems Inc. (Türkiye) using coded samples.

### 2.7. Statistical Analysis

Statistical analyses were conducted using IBM SPSS Statistics version 27.0 (IBM Corp., Armonk, NY, USA). The revised Spearman correlation analyses and Benjamini–Hochberg false discovery rate (FDR) correction were performed using Python version 3.13.5 (Python Software Foundation, Beaverton, OR, USA), with SciPy version 1.17.0 (SciPy project) and statsmodels version 0.14.6. Data are presented as mean ± standard deviation (SD), with 95% confidence intervals where appropriate. Normality was assessed using the Shapiro–Wilk test, and homogeneity of variance was evaluated using Levene’s test.

For normally distributed variables with homogeneous variances, differences among the four experimental groups were analyzed using one-way analysis of variance followed by Tukey’s post hoc test. When homogeneity of variance was not satisfied, Welch’s ANOVA followed by the Games–Howell post hoc test was used. Non-normally distributed variables were analyzed using the Kruskal–Wallis test followed by Dunn’s multiple-comparison test with Bonferroni adjustment.

Male and female offspring were analyzed separately to characterize response patterns within each sex. No formal statistical comparison between sexes was performed. Within each sex, two-way factorial ANOVA was used to assess the main effects of maternal diet (standard chow vs. cafeteria diet), maternal propolis supplementation (absent vs. present), and the maternal diet × propolis interaction. Partial eta squared (η_p_^2^) was reported as the effect-size measure. No single primary endpoint was designated a priori because the study was designed to assess a prespecified set of outcomes across complementary metabolic, inflammatory, histopathological, and transcriptional domains. The study is therefore interpreted as a hypothesis-driven, multi-endpoint preclinical investigation rather than as a confirmatory study centered on a single primary outcome.

For outcomes showing a significant maternal diet × propolis interaction, simple effects of propolis were examined within each maternal diet stratum using estimated marginal means with pairwise comparisons between CONP and CON and between CAFP and CAF. Estimated mean differences and 95% confidence intervals were reported together with Hedges’ g effect sizes and their 95% confidence intervals. For female triglycerides, for which the homogeneity-of-variance assumption was not satisfied, Welch’s unequal-variance tests were additionally performed as sensitivity analyses for the within-diet contrasts.

Given the number of outcomes evaluated, individual nominal *p* values were interpreted together with effect sizes, consistency across related endpoints, and the overall biological pattern rather than as isolated confirmatory findings. Accordingly, particular caution was applied to marginally significant and endpoint-specific findings.

No formal a priori power calculation was performed. The planned sample size was *n* = 8 dams per maternal treatment group, corresponding to a maximum of *n* = 8 offspring per sex/group because only one offspring of each sex was selected from each litter. Unless otherwise specified, analyses were conducted with *n* = 8 per group or *n* = 8 per sex/group for offspring analyses. For relative organ-weight analyses, one male offspring in the CAF group could not be included because terminal body weight was unavailable, precluding calculation of relative organ weight; therefore, these analyses included *n* = 7 for CAF males. Among dams, liver and right and left kidney weight measurements were unavailable for one animal in the CONP group, resulting in *n* = 7 for the corresponding relative organ-weight analyses, whereas the relative heart-weight analysis included *n* = 8 per group. No missing values were imputed, and analyses were performed using the available observations.

Spearman rank-correlation analyses were conducted separately in male and female offspring to examine relationships among biochemical, histopathological, and hepatic gene-expression variables. Pairwise complete observations were used. The correlation matrix comprised 17 variables, corresponding to 136 unique pairwise correlations within each sex. To account for multiple testing, the resulting *p* values were adjusted separately within male and female offspring using the Benjamini–Hochberg false discovery rate (FDR) procedure. FDR-adjusted q values < 0.05 were considered statistically significant for the correlation analyses. Because these analyses were exploratory, the resulting associations were interpreted as hypothesis-generating and not as evidence of causal or mechanistic relationships. For analyses other than the correlation analysis, statistical significance was set at *p* < 0.05. For the exploratory correlation analyses, statistical significance was evaluated using Benjamini–Hochberg FDR-adjusted q values, with q < 0.05 considered statistically significant. The study protocol was retrospectively registered on the Open Science Framework (OSF; https://osf.io/tc39u/ registered on 9 April 2026; accessed on 28 August 2026).

## 3. Results

### 3.1. Maternal Dietary Intake During the Intervention Period

Maternal energy and nutrient intakes across the 14-week maternal intervention period, spanning preconception, gestation, and lactation, are presented in Table 1. Mean daily energy intake was significantly higher in the CAF and CAFP groups than in the CON and CONP groups (*p* < 0.001). A similar pattern was observed for carbohydrate intake, with higher values in the CAF and CAFP groups than in both standard-chow groups (*p* < 0.001). Protein intake did not differ significantly among the groups (*p* = 0.798). Fat intake was significantly higher in the CAF and CAFP groups than in the CON and CONP groups (*p* < 0.001). Fiber intake also differed significantly among the groups (*p* < 0.001); the CAF group had a lower intake than the CON and CONP groups, whereas the CAFP group showed an intermediate value that did not differ significantly from the other groups. Sodium intake was significantly higher in the CAF and CAFP groups than in the CON and CONP groups (*p* < 0.001).

### 3.2. Final Body Weight and Relative Organ Weights in Dams and Offspring

Final body weight did not differ significantly among experimental groups in dams or in male or female offspring (all (*p* > 0.05)). Relative liver, heart, and kidney weights are presented in Table 2.

In dams, relative liver weight differed among groups (*p* = 0.015); CAFP dams had a higher relative liver weight than CAF and CONP dams. Relative heart and right and left kidney weights also differed among groups (all (*p* < 0.001)). CONP dams had a higher relative heart weight than CAF and CAFP dams, while relative kidney weights were generally highest in CONP dams. These findings did not demonstrate a uniform cafeteria-diet effect or a consistent normalization by propolis across all maternal organs.

In male offspring, relative liver weight did not differ among groups (*p* = 0.545). Relative heart, right kidney, and left kidney weights differed significantly ((*p* = 0.002), (*p* = 0.006), and (*p* = 0.020), respectively). CAFP males had a lower relative heart weight than CON and CONP males, whereas CAF males had lower relative right and left kidney weights than CONP males.

In female offspring, relative liver and heart weights differed among groups ((*p* = 0.017) and (*p* = 0.011), respectively). Relative liver weight was lower in CAFP than in CON offspring, and relative heart weight was lower in CAFP than in CONP offspring. Although the omnibus test for relative right kidney weight was significant (*p* = 0.040), no Tukey-adjusted pairwise comparison reached significance. Relative left kidney weight did not differ among groups (*p* = 0.570).

### 3.3. Metabolic and Inflammatory Profiles in Adult Offspring

**Figure 2:**
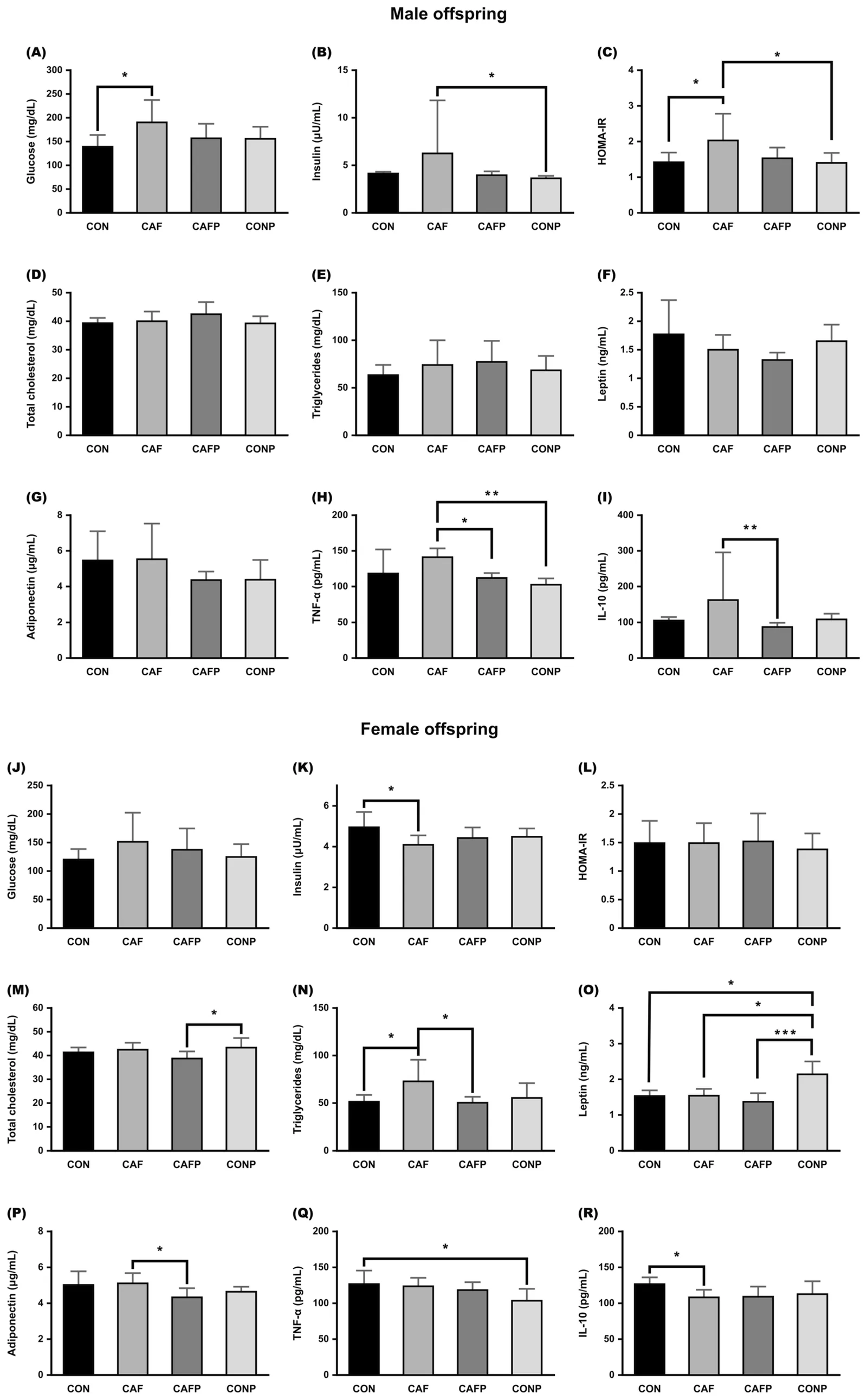
Serum metabolic, adipokine, and inflammatory profiles of male and female offspring at 18 weeks of age. Panels show glucose (**A**,**J**), insulin (**B**,**K**), homeostatic model assessment of insulin resistance (HOMA-IR; (**C**,**L**)), total cholesterol (**D**,**M**), triglycerides (**E**,**N**), leptin (**F**,**O**), adiponectin (**G**,**P**), tumor necrosis factor-alpha (TNF-α; (**H**,**Q**)), and interleukin-10 (IL-10; (**I**,**R**)) concentrations in male (**A**–**I**) and female (**J**–**R**) offspring. Data are presented as mean ± standard deviation (SD; *n* = 8/sex/group). Group differences were evaluated using one-way analysis of variance followed by Tukey’s post hoc test or the Kruskal–Wallis test followed by Dunn’s test with Bonferroni correction, as appropriate. Horizontal brackets indicate significant pairwise differences: * *p* < 0.05, ** *p* < 0.01, and *** *p* < 0.001. Graphs were generated using GraphPad Prism version 9 (GraphPad Software, Boston, MA, USA), and the composite figure was assembled by the authors using Inkscape (version 1.4.3). Abbreviations: CON, standard chow and drinking water; CONP, standard chow and propolis in drinking water; CAF, cafeteria diet and drinking water; CAFP, cafeteria diet and propolis in drinking water; HOMA-IR, homeostatic model assessment of insulin resistance; TNF-α, tumor necrosis factor-alpha; IL-10, interleukin-10.

The metabolic and inflammatory profiles of male and female offspring are presented in Figure 2.

In male offspring, significant group effects were detected for glucose (*p* = 0.039), insulin (*p* = 0.035), and HOMA-IR (*p* = 0.029). Glucose was higher in CAF than in CON offspring. Insulin was higher in CAF than in CONP offspring, while HOMA-IR was higher in CAF than in both CON and CONP offspring. Total cholesterol, triglycerides, leptin, and adiponectin did not differ significantly among groups (all (*p* > 0.05)). TNF-α and IL-10 both showed significant group effects ((*p* = 0.003) for each). TNF-α was higher in CAF than in CAFP and CONP offspring, whereas IL-10 was higher in CAF than in CAFP offspring.

In female offspring, glucose and HOMA-IR did not differ among groups ((*p* = 0.324) and (*p* = 0.898), respectively), whereas insulin differed significantly (*p* = 0.026), with higher concentrations in CON than in CAF offspring. Total cholesterol did not show a significant overall group effect (*p* = 0.113). Triglycerides differed among groups (*p* = 0.015) and were higher in CAF than in CON and CAFP offspring. Leptin differed markedly among groups (*p* < 0.001), with higher concentrations in CONP than in CON, CAF, and CAFP offspring. Adiponectin was higher in CAF than in CAFP offspring (*p* = 0.032). TNF-α was higher in CON than in CONP offspring (*p* = 0.021), while IL-10 was higher in CON than in CAF offspring (*p* = 0.038).

These sex-stratified analyses showed different patterns of statistically significant findings in males and females; however, because sex was not included as a factor in a combined model, these patterns should not be interpreted as formal evidence of a sex difference.

### 3.4. Hepatic Histopathology in Dams and Offspring

**Figure 3:**
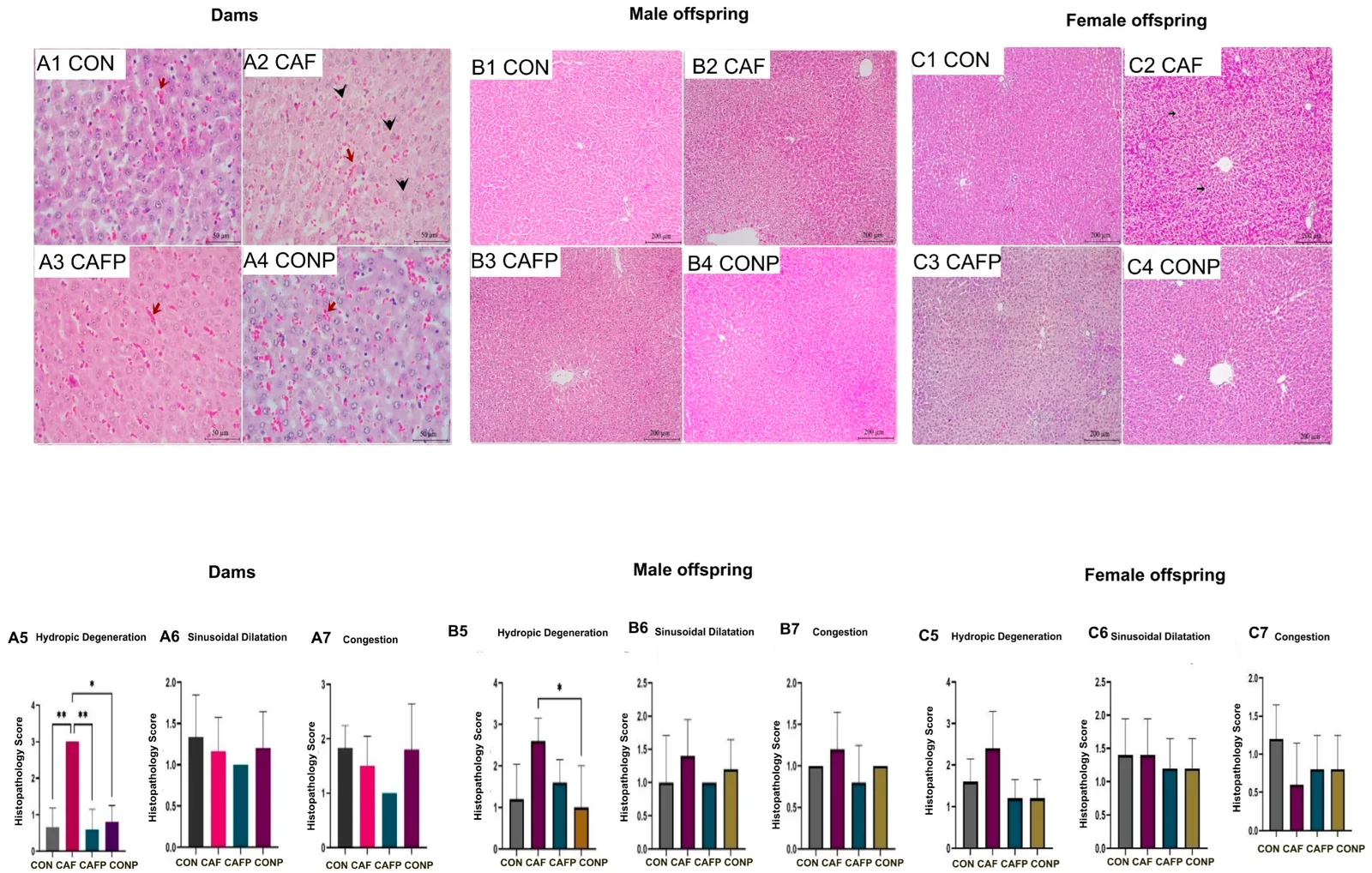
Representative liver histology and semiquantitative histopathological scores in dams (**A1**–**A7**), male offspring (**B1**–**B7**), and female offspring (**C1**–**C7**). Panels (**A1**–**A4**), (**B1**–**B4**), and (**C1**–**C4**) show representative H&E-stained liver sections for CON, CAF, CAFP, and CONP, respectively, whereas panels (**A5**–**A7**), (**B5**–**B7**), and (**C5**–**C7**) show the semiquantitative histopathological scores for hydropic degeneration, sinusoidal dilatation, and congestion, respectively.Red arrows indicate representative areas of hydropic degeneration, whereas black arrowheads indicate sinusoidal alterations and/or congestion. Higher scores indicate greater histopathological severity. Dam liver samples were collected at the end of lactation, and offspring liver samples were collected at 18 weeks of age. Data are presented as mean ± standard deviation (SD; *n* = 8/group). Group differences were evaluated using the Kruskal–Wallis test followed by Dunn’s multiple-comparison test with Bonferroni correction. Horizontal brackets indicate significant pairwise differences: * *p* < 0.05 and ** *p* < 0.01. Scale bars: 50 μm for dam sections and 200 μm for offspring sections. Graphs were generated using GraphPad Prism, and the composite figure was assembled by the authors using Inkscape (version 1.4.3). Abbreviations: CON, standard chow and drinking water; CONP, standard chow and propolis in drinking water; CAF, cafeteria diet and drinking water; CAFP, cafeteria diet and propolis in drinking water.

Representative liver sections and semi-quantitative histopathology scores are presented in Figure 3.

In dams, hepatocellular hydropic degeneration was higher in CAF than in CON and CAFP dams (both adjusted (*p* < 0.01)) and CONP dams (adjusted (*p* < 0.05)). Thus, a direct CAF–CAFP difference was detected for maternal hydropic degeneration. Sinusoidal dilatation and congestion scores did not differ significantly among maternal groups.

In male offspring, hydropic degeneration was higher in CAF than in CONP offspring (adjusted (*p* < 0.05)). No significant CAF–CAFP difference was detected; therefore, the male histopathology findings do not provide direct statistical evidence of attenuation by propolis. Sinusoidal dilatation and congestion did not differ significantly among male groups.

In female offspring, hydropic degeneration, sinusoidal dilatation, and congestion scores did not differ significantly among groups (all (*p* > 0.05)).

### 3.5. Hepatic Expression of Lipid-Metabolism-Related Genes

**Figure 4:**
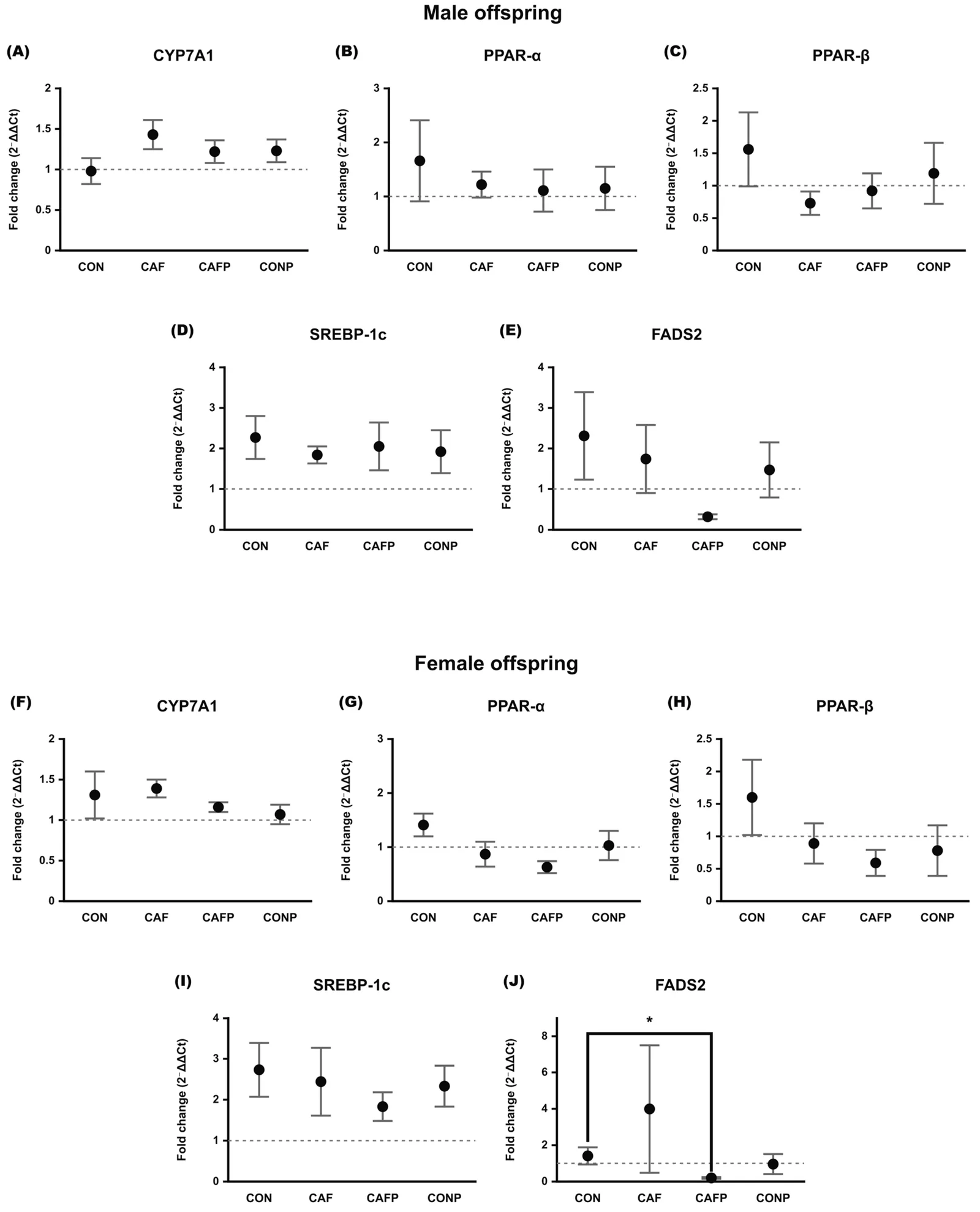
Hepatic expression of lipid-metabolism-related genes in adult male and female offspring. Hepatic mRNA expression levels of CYP7A1 (**A**,**F**), PPAR-α (**B**,**G**), PPAR-β (**C**,**H**), SREBP-1c (**D**,**I**), and FADS2 (**E**,**J**) are shown for male (**A**–**E**) and female (**F**–**J**) offspring at 18 weeks of age. Gene expression was normalized to β-actin and calculated using the 2^−ΔΔCt^ method. The dashed horizontal line represents a fold-change value of 1. Data are presented as mean ± standard error of the mean (SEM; n=8/sex/group). Group differences were evaluated using one-way ANOVA followed by Tukey’s post hoc test or Welch’s ANOVA followed by the Games–Howell test, as appropriate. The horizontal bracket indicates a significant pairwise difference, where * *p* < 0.05. Graphs were generated using GraphPad Prism, and the composite figure was assembled by the authors using Inkscape (version 1.4.3). Abbreviations: CON, standard chow and drinking water; CONP, standard chow and propolis in drinking water; CAF, cafeteria diet and drinking water; CAFP, cafeteria diet and propolis in drinking water.

Hepatic mRNA expression levels of CYP7A1, PPAR-α, PPAR-β, SREBP-1c, and FADS2 are shown in Figure 4.

In male offspring, none of the five genes differed significantly among groups (all (*p* > 0.05)). FADS2 showed a borderline overall group effect (*p* = 0.051), but no statistically significant post hoc comparison was identified. Accordingly, no consistent transcriptional restoration by maternal propolis supplementation was demonstrated in male offspring.

In female offspring, CYP7A1, PPAR-β, and SREBP-1c did not show significant one-way group effects (all (*p* > 0.05)). The PPAR-α group effect was borderline (*p* = 0.050), without a significant adjusted pairwise difference. FADS2 expression differed significantly among groups (*p* = 0.034), with lower expression in CAFP than in CON offspring. Therefore, the FADS2 result reflected reduced—not increased—expression in the CAFP group.

### 3.6. Sex-Stratified Factorial Effects of Maternal Diet and Propolis Supplementation

**Figure 5:**
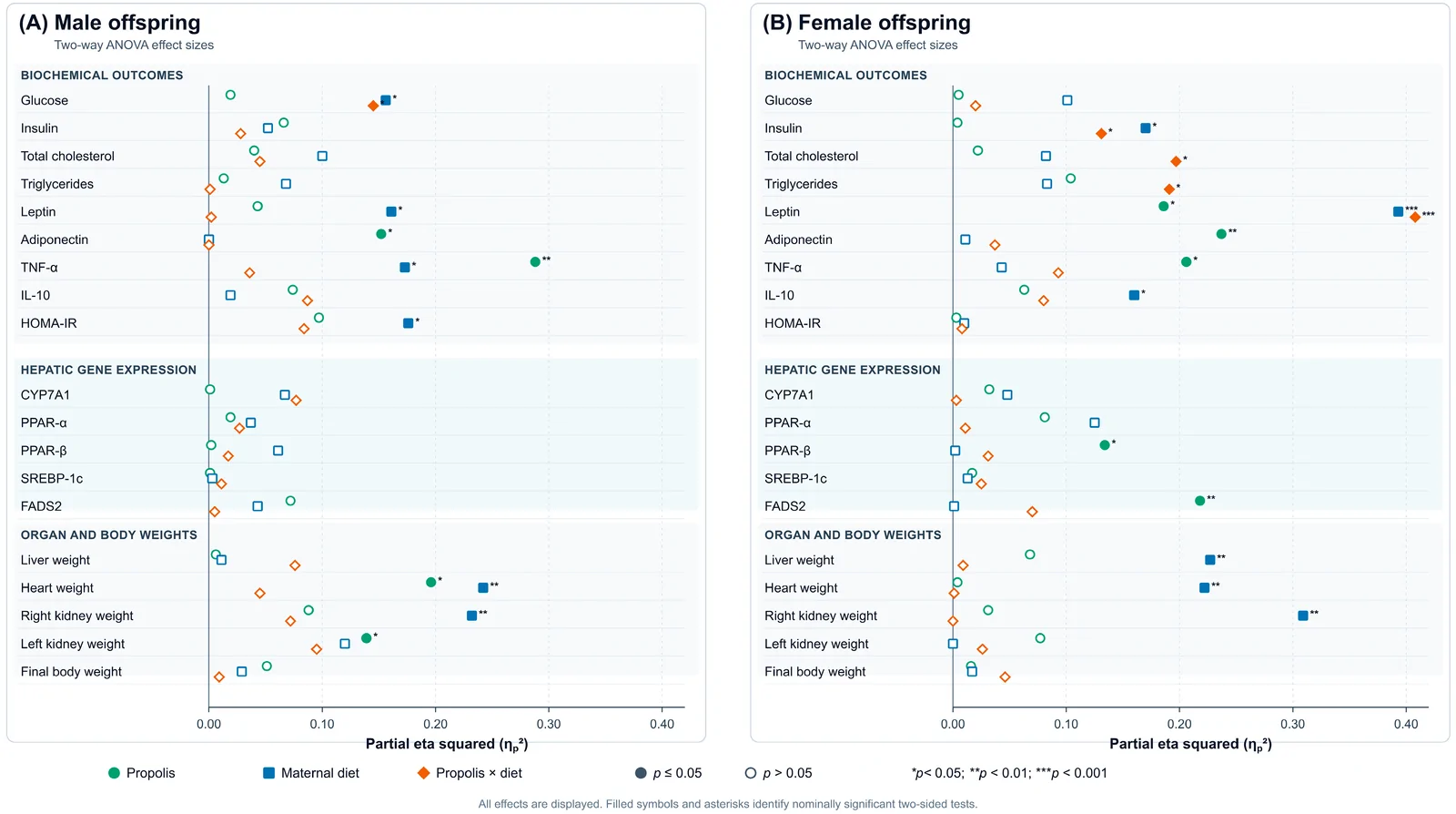
Sex-stratified effect sizes of maternal diet, propolis supplementation, and their interaction on offspring outcomes. Partial eta-squared ($\eta_{p}^{2}$) values obtained from sex-stratified two-way ANOVA models are presented for male (**A**) and female (**B**) offspring. Outcomes are grouped into biochemical variables, hepatic gene-expression markers, and relative organ and final body weights. Green circles represent the main effect of propolis supplementation, blue squares represent the main effect of maternal diet, and orange diamonds represent the maternal diet × propolis interaction. Filled symbols and accompanying asterisks indicate statistically significant effects, whereas open symbols indicate non-significant effects. Larger $\eta_{p}^{2}$ values indicate a greater proportion of variance explained by the corresponding factor but do not indicate the direction of the effect. All model-derived effects are displayed. Complete factorial ANOVA statistics are provided in Supplementary Tables S4 and S5. Within-diet contrasts for outcomes showing significant maternal diet × propolis interactions are provided in Supplementary Table S6. Abbreviations: HOMA-IR, homeostatic model assessment of insulin resistance; TNF-α, tumor necrosis factor-alpha; IL-10, interleukin-10; $\eta_{p}^{2}$, partial eta-squared.

The magnitudes of the maternal-diet, propolis, and diet × propolis effects obtained from sex-stratified two-way ANOVA models are summarized in Figure 5.

In male offspring, significant maternal-diet effects were identified for glucose ((*p* = 0.034), η_p_^2^ = 0.156), leptin ((*p* = 0.028), η_p_^2^ = 0.161), TNF-α ((*p* = 0.022), η_p_^2^ = 0.173), HOMA-IR ((*p* = 0.023), η_p_^2^ = 0.176), relative heart weight ((*p* = 0.006), η_p_^2^ = 0.242), and relative right kidney weight ((*p* = 0.007), η_p_^2^ = 0.232). Significant propolis effects were found for adiponectin ((*p* = 0.033), η_p_^2^ = 0.152), TNF-α ((*p* = 0.002), η_p_^2^ = 0.288), relative heart weight ((*p* = 0.014), η_p_^2^ = 0.196), and relative left kidney weight ((*p* = 0.042), η_p_^2^ = 0.139). A diet × propolis interaction was detected for glucose ((*p* = 0.042), η_p_^2^ = 0.145).

In female offspring, significant maternal-diet effects were found for insulin ((*p* = 0.023), η_p_^2^ = 0.170), leptin ((*p* < 0.001), η_p_^2^ = 0.393), IL-10 ((*p* = 0.028), η_p_^2^ = 0.160), relative liver weight ((*p* = 0.008), η_p_^2^ = 0.227), relative heart weight ((*p* = 0.009), η_p_^2^ = 0.222), and relative right kidney weight ((*p* = 0.001), η_p_^2^ = 0.309). Significant propolis effects were detected for leptin ((*p* = 0.017), η_p_^2^ = 0.186), adiponectin ((*p* = 0.006), η_p_^2^ = 0.237), TNF-α ((*p* = 0.012), η_p_^2^ = 0.206), PPAR-β expression ((*p* = 0.046), η_p_^2^ = 0.134), and FADS2 expression ((*p* = 0.009), η_p_^2^ = 0.218). Significant diet × propolis interactions were observed for insulin ((*p* = 0.049), η_p_^2^ = 0.131), total cholesterol ((*p* = 0.014), η_p_^2^ = 0.197), triglycerides ((*p* = 0.016), η_p_^2^ = 0.191), and leptin ((*p* < 0.001), η_p_^2^ = 0.408).

For outcomes showing a significant maternal diet × propolis interaction, within-diet contrasts were examined to clarify the source of the interaction (Supplementary Table S6). In male offspring, neither the CONP versus CON glucose contrast (*p* = 0.317) nor the CAFP versus CAF contrast reached statistical significance, although glucose was lower in CAFP than CAF (mean difference = −33.457 mg/dL, 95% CI −68.028 to 1.115; *p* = 0.057). In female offspring, neither within-diet contrast for insulin was significant (CONP vs. CON, *p* = 0.100; CAFP vs. CAF, *p* = 0.237). For total cholesterol, CAFP had a significantly lower concentration than CAF (mean difference = −3.720 mg/dL, 95% CI −6.882 to −0.558; *p* = 0.023; Hedges’ g = −1.186, 95% CI −2.193 to −0.145), whereas CONP did not differ significantly from CON (*p* = 0.207). For triglycerides, CAFP likewise had a lower concentration than CAF (mean difference = −22.508 mg/dL, 95% CI −37.394 to −7.621; *p* = 0.004; Hedges’ g = −1.280, 95% CI −2.301 to −0.223), whereas the CONP versus CON contrast was not significant (*p* = 0.590). Because variance homogeneity was not satisfied for triglycerides, Welch sensitivity analyses were additionally performed and supported the same interpretation (CAFP vs. CAF, *p* = 0.027; CONP vs. CON, *p* = 0.528). For leptin, CONP had a significantly higher concentration than CON (mean difference = 0.611 ng/mL, 95% CI 0.355 to 0.867; *p* < 0.001; Hedges’ g = 2.069, 95% CI 0.849 to 3.244), whereas CAFP did not differ significantly from CAF (*p* = 0.199).

### 3.7. Exploratory Multidomain Correlation Analysis

**Figure 6:**
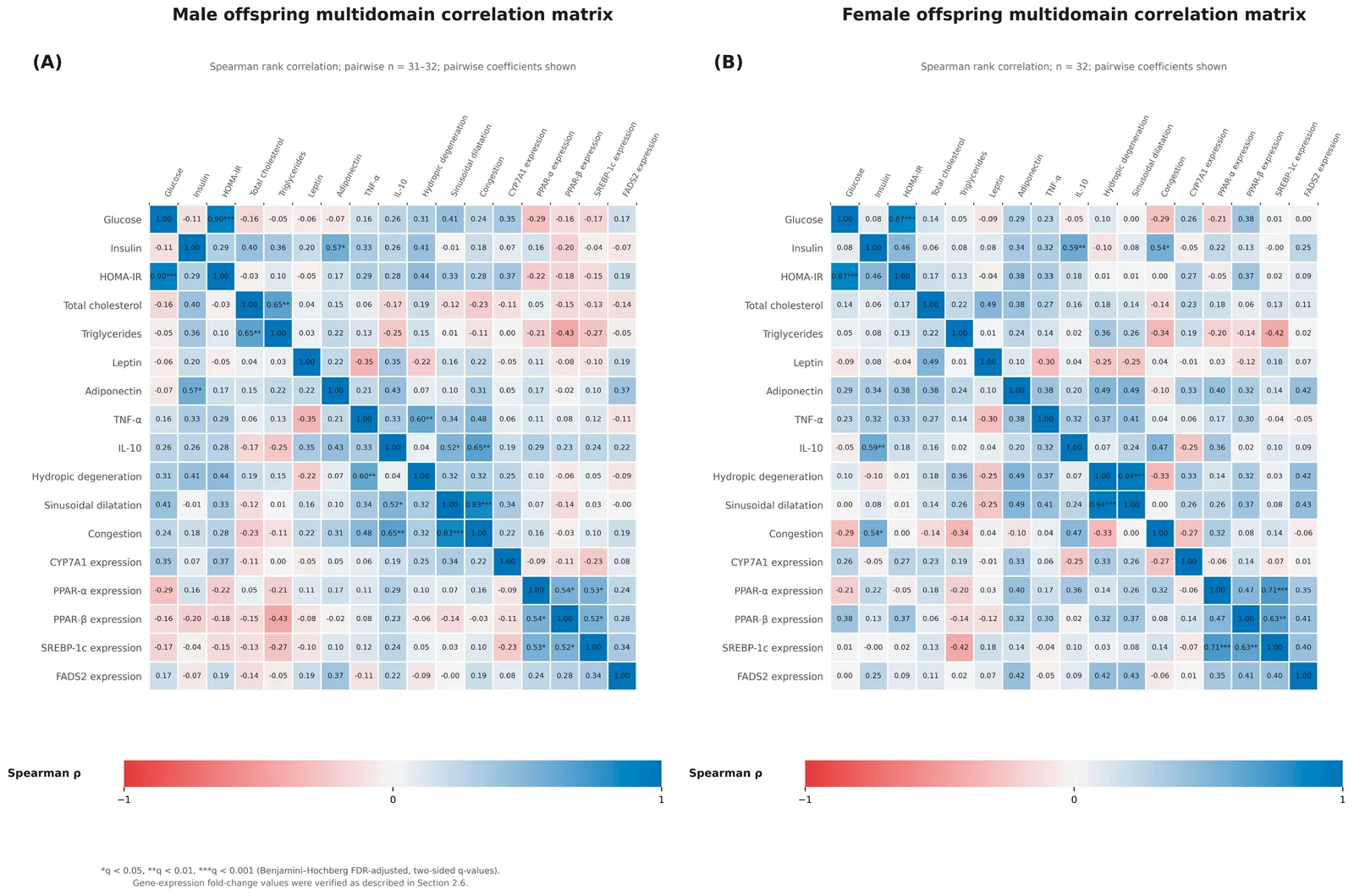
Sex-stratified multidomain correlation matrices in adult offspring. Spearman rank-correlation matrices are presented for male (**A**) and female (**B**) offspring at 18 weeks of age. The matrices include biochemical and inflammatory markers (glucose, insulin, HOMA-IR, total cholesterol, triglycerides, leptin, adiponectin, TNF-α, and IL-10), hepatic histopathological scores (hydropic degeneration, sinusoidal dilatation, and congestion), and hepatic gene-expression outcomes (CYP7A1, PPAR-α, PPAR-β, SREBP-1c, and FADS2). Each cell displays Spearman’s correlation coefficient (ρ). Pairwise sample sizes were 31–32 for males and 32 for females. Gene-expression fold-change values used in the correlation analyses were verified as described in Section 2.6. Multiple testing was controlled separately within each sex using the Benjamini–Hochberg false discovery rate procedure across the 136 unique pairwise correlations.Abbreviations: HOMA-IR, homeostatic model assessment of insulin resistance; TNF-α, tumor necrosis factor-alpha; IL-10, interleukin-10.

Sex-stratified Spearman correlation matrices were generated to examine associations among biochemical, inflammatory, histopathological, and hepatic gene-expression outcomes (Figure 6). Pairwise sample sizes were 31–32 in males and 32 in females. Maternal nutrient-intake variables were not included because the matrices were restricted to multidomain outcomes measured directly in adult offspring. To account for multiple testing, the 136 unique pairwise correlations within each sex were adjusted separately using the Benjamini–Hochberg false discovery rate procedure, and associations with FDR-adjusted q < 0.05 were considered statistically significant.

In male offspring, 10 associations remained statistically significant after FDR correction. Glucose was strongly correlated with HOMA-IR (Spearman’s ρ = 0.90, q < 0.001), and sinusoidal dilatation was strongly correlated with congestion (ρ = 0.83, q < 0.001). Additional positive associations were observed between IL-10 and congestion (ρ = 0.65, q = 0.0025), total cholesterol and triglycerides (ρ = 0.65, q = 0.0026), TNF-α and hydropic degeneration (ρ = 0.60, q = 0.0079), insulin and adiponectin (ρ = 0.57, q = 0.0154), and IL-10 and sinusoidal dilatation (ρ = 0.52, q = 0.0346). Within the hepatic gene-expression domain, PPAR-α was positively correlated with PPAR-β (ρ = 0.54, q = 0.0286) and SREBP-1c (ρ = 0.53, q = 0.0295), while PPAR-β was positively correlated with SREBP-1c (ρ = 0.52, q = 0.0308).

In female offspring, six associations remained statistically significant after FDR correction. Hydropic degeneration was strongly correlated with sinusoidal dilatation (ρ = 0.94, q < 0.001), and glucose was strongly correlated with HOMA-IR (ρ = 0.87, q < 0.001). Among hepatic transcriptional markers, PPAR-α was positively correlated with SREBP-1c (ρ = 0.71, q < 0.001), and PPAR-β was positively correlated with SREBP-1c (ρ = 0.63, q = 0.0034). Insulin was also positively associated with IL-10 (ρ = 0.59, q = 0.0096) and congestion (ρ = 0.54, q = 0.0315). Although these associations remained significant after FDR correction, the correlation analyses were exploratory and do not establish causal or mechanistic relationships.

## 4. Discussion

The present study examined whether maternal propolis supplementation modified the long-term consequences of cafeteria-diet exposure in separately analyzed male and female offspring. Maternal cafeteria feeding markedly increased the intake of energy, carbohydrate, fat, and sodium. Nevertheless, final body weight did not differ among groups in dams or offspring. In the male analysis, notable findings involved glucose regulation, inflammatory markers, and hepatic histopathology, whereas the female analysis showed a heterogeneous pattern involving lipid-related and adipokine outcomes. These observations describe the separate sex-stratified analyses and should not be interpreted as formal evidence of a sex effect. Maternal propolis supplementation modified selected outcomes, but these effects were neither uniform across biological domains nor consistently restorative. Thus, the overall findings support selective, endpoint-specific, and context-dependent modulation by propolis rather than generalized metabolic or hepatic protection. The elevated energy and macronutrient intake observed in CAF and CAFP dams confirms the ability of the cafeteria model to produce a markedly obesogenic nutritional exposure. Akyol et al. used a rotating selection of palatable foods during pregnancy and showed that the cafeteria model produced a nutritionally unbalanced maternal dietary environment that differed substantially from standard chow feeding [13]. The present intake data are consistent with this feature of the model.

Oliva et al. reported that the palatability and variety of cafeteria foods, rather than dietary fat content alone, were important drivers of increased food consumption [31]. This observation may explain why the CAF and CAFP dams in the present study consumed substantially more energy despite continued access to standard chow.

Sampey et al. directly compared cafeteria feeding with a conventional high-fat diet in adult Wistar rats and found that cafeteria feeding produced greater hyperphagia, glucose dysregulation, systemic inflammation, and hepatic steatosis [32]. Although their experiment involved direct exposure of adult animals rather than maternal developmental programming, it supports the metabolic potency of the cafeteria-diet model used in the present study.

Importantly, propolis supplementation did not prevent the elevated energy intake associated with cafeteria feeding. CAF and CAFP dams showed similarly high intake levels, indicating that the offspring effects associated with propolis cannot readily be attributed to reduced maternal energy consumption. Accordingly, any propolis-related differences should be interpreted as selective biological modulation rather than as a consequence of lower exposure to the cafeteria diet.

Final body weight was comparable across groups, but several relative organ weights differed. These changes were not uniformly corrected by propolis. For example, some kidney-weight differences were less marked in propolis-exposed groups, whereas other outcomes, particularly relative heart weight, did not demonstrate a consistent protective pattern. Relative organ weights are influenced by both the organ-weight numerator and the body-weight denominator and should therefore not be interpreted as direct evidence of organ injury without corresponding histological or functional findings.

An important feature of the present study is that metabolic and hepatic alterations were observed despite comparable final body weights among offspring groups. This finding suggests that developmental programming may manifest through metabolic and tissue-specific alterations without necessarily being accompanied by detectable differences in overall body mass at the time of assessment. A similar dissociation between body-weight gain and metabolic dysfunction has been reported by Giudetti et al., who demonstrated that cafeteria-diet exposure impaired hepatic metabolic regulation even in the absence of excessive body-weight gain [33]. Evidence from maternal cafeteria-diet models also supports the possibility of tissue-specific programming that may not be adequately reflected by overall body weight. Santos et al. reported persistent tissue-specific morphological alterations in adult male offspring following maternal cafeteria-diet exposure [15], while Vithayathil et al. demonstrated marked effects of maternal cafeteria-diet exposure on offspring fat deposition and adipose-tissue SREBP-1c expression [16]. Collectively, these findings indicate that body weight alone may not fully capture the metabolic and tissue-specific consequences of early-life nutritional programming. Nevertheless, because body composition and adiposity were not directly assessed in the present study, our findings should be interpreted as metabolic and hepatic alterations occurring in the absence of detectable differences in final body weight rather than as evidence for the complete absence of an obesity-related phenotype.

Within the male offspring analysis, glucose-related outcomes were among the principal metabolic findings. The CAF group had the highest mean glucose, insulin, and HOMA-IR values. The factorial analysis identified significant maternal-diet effects on glucose and HOMA-IR, together with a maternal diet × propolis interaction for glucose. However, the subsequent within-diet contrasts showed that neither CONP versus CON nor CAFP versus CAF reached statistical significance. Although glucose was numerically lower in CAFP than CAF, the corresponding contrast was marginal and did not reach the prespecified significance threshold (*p* = 0.057). Therefore, the significant interaction indicates that the association of propolis with glucose differed according to maternal dietary background, but it does not provide sufficient evidence that propolis significantly attenuated the cafeteria-associated glucose alteration. Likewise, no significant propolis main effect was detected for insulin or HOMA-IR. Collectively, these findings support an effect of maternal cafeteria exposure on glycemic regulation in male offspring but do not demonstrate normalization of glucose homeostasis by maternal propolis supplementation. Akyol et al. showed that glucose intolerance after maternal cafeteria feeding was strongly modified by the offspring’s post-weaning diet [34]. In the present study, all offspring consumed standard chow after weaning, thereby removing post-weaning dietary exposure as a source of group variation. The persistence of male glucose-related differences under these standardized conditions suggests that maternal exposure retained a detectable influence into adulthood. However, the differences between the two studies also indicate that the magnitude and persistence of the phenotype may depend on post-weaning nutrition, age at assessment, and the duration of maternal exposure.

In male offspring, CAF exposure was accompanied by higher TNF-α and IL-10 concentrations. The simultaneous elevation of a pro-inflammatory cytokine and a counter-regulatory cytokine is more appropriately interpreted as altered inflammatory regulation than as a uniformly pro-inflammatory response. Propolis showed a significant main effect on TNF-α, and CAFP males had lower TNF-α concentrations than CAF males. Nevertheless, the IL-10 pattern did not demonstrate equivalent normalization.

Matuszewska et al. reported increased circulating IL-6, IL-10, and TNF-α concentrations in young offspring exposed to a maternal cafeteria diet [17]. Their simultaneous observation of pro- and anti-inflammatory cytokine changes supports the interpretation that maternal cafeteria feeding can alter the balance of inflammatory regulation. Their study reported greater susceptibility in female offspring at an earlier developmental stage. Direct comparison with the present sex-stratified findings is limited because sex was not included as a factor in our statistical models, and the studies also differed in assessment age and endpoints. Within the female offspring analysis, insulin differed among groups and showed both a maternal-diet effect and a maternal diet × propolis interaction, whereas glucose and HOMA-IR did not show corresponding alterations. Importantly, the within-diet contrasts clarified that the insulin interaction was not attributable to a statistically significant propolis effect within either dietary background. In contrast, the interactions for total cholesterol and triglycerides were accompanied by significantly lower concentrations in CAFP than CAF, whereas the corresponding CONP versus CON contrasts were not significant. The triglyceride finding was also retained in the Welch sensitivity analysis performed because of variance heterogeneity. These findings provide evidence that the association of maternal propolis exposure with selected lipid outcomes depended on the cafeteria-diet background. The leptin interaction showed a different pattern: leptin was significantly higher in CONP than CON, whereas CAFP did not differ significantly from CAF. Thus, the leptin interaction cannot be interpreted as attenuation of a cafeteria-diet effect. Taken together, the female findings indicate outcome-specific and diet-context-dependent modulation rather than a uniform metabolic response to propolis.

Choi reported that maternal and post-weaning high-fat feeding altered leptin sensitivity and hypothalamic appetite-regulatory pathways in offspring [35]. These findings demonstrate that early obesogenic exposure can produce persistent leptin dysregulation. However, because the present leptin response depended on both maternal diet and propolis and was highest in CONP females, the current findings suggest a more complex adipokine response than a simple cafeteria-induced increase.

The sex-stratified factorial analyses further support this distinction. Maternal-diet effects in males were concentrated in glucose, HOMA-IR, leptin, TNF-α, and selected organ weights. In females, maternal-diet effects were detected for insulin, leptin, IL-10, and several organ weights. Propolis effects in females included leptin, adiponectin, TNF-α, PPAR-β, and FADS2, whereas interactions were evident for insulin, total cholesterol, triglycerides, and leptin. These results indicate that propolis did not exert a single diet-independent effect; instead, its associations varied across outcomes and maternal dietary backgrounds within the separate male and female analyses.

Hydropic degeneration was the principal histopathological alteration observed in liver tissue. CAF dams showed greater hydropic degeneration than the other maternal groups, whereas sinusoidal dilatation and congestion did not differ significantly. Because steatosis, fibrosis, collagen deposition, and hepatic lipid accumulation were not assessed, these findings should be interpreted only within the histopathological endpoints evaluated. Male offspring exposed to maternal CAF feeding also showed increased hydropic degeneration, but the significant pairwise difference was observed between CAF and CONP. CAFP males had an intermediate score and did not differ significantly from CAF in the reported pairwise analysis. Accordingly, the male histopathological findings do not provide direct statistical evidence that maternal propolis supplementation attenuated hydropic degeneration.

Santos et al. reported that lifelong maternal cafeteria exposure produced persistent liver and adipose-tissue morphological changes in adult male offspring [15]. Their findings provide relevant context for the histopathological alterations observed in male offspring in the present study, although the dietary composition, exposure design, and histological endpoints were not identical.

Female offspring showed no significant group differences in hydropic degeneration, sinusoidal dilatation, or congestion. Gasparin et al. found that female mice directly exposed to a cafeteria diet developed more extensive hepatic steatosis than males despite comparable body weight and adiposity [14]. Because Gasparin et al. directly compared males and females, whereas the present study analyzed the two sexes separately, the studies are not directly comparable with respect to sex effects. In addition, the offspring themselves consumed the cafeteria diet in their study, whereas exposure in the present study was restricted to the maternal period and all offspring received standard chow after weaning. The predominance of hydropic degeneration, together with the absence of group differences in sinusoidal dilatation and congestion, indicates that the detected histopathological differences were limited to the endpoints assessed. No inference regarding steatosis, fibrosis, collagen deposition, hepatic lipid accumulation, or overall liver-disease severity can be made because these features were not evaluated. Hepatic expression of CYP7A1, PPAR-α, PPAR-β, SREBP-1c, and FADS2 did not differ significantly among male groups. Accordingly, the male findings do not support broad or persistent transcriptional remodeling of these lipid-metabolism pathways at 18 weeks of age, despite the presence of biochemical and histopathological alterations.

Vithayathil et al. found that exposure to a maternal cafeteria diet during the suckling period had a particularly strong effect on offspring fat deposition and adipose-tissue SREBP-1c expression [16]. The absence of a significant hepatic SREBP-1c difference in the adult offspring in the present study may indicate that transcriptional responses observed in other metabolic tissues or at earlier ages do not necessarily persist in the adult liver. Differences in the tissue examined, exposure timing, age at sample collection, and post-weaning diet may also account for the contrasting results.

In females, the conventional four-group analysis identified FADS2 as the only gene with a significant overall group difference, with lower expression in CAFP than in CON, while the CAF estimate was highly variable. The factorial analysis additionally identified significant propolis main effects for PPAR-β and FADS2. However, these findings represent limited transcriptional signals within a small targeted gene panel and should not be interpreted as evidence of broad regulation of hepatic lipid-metabolism pathways. In particular, PPAR-β did not show a significant overall difference in the conventional four-group analysis, and the FADS2 difference reflected lower—not restored—expression in CAFP. The biological significance of both findings therefore remains uncertain. Because FADS2 participates in long-chain polyunsaturated fatty-acid synthesis, the observed mRNA difference may be relevant to hepatic fatty-acid handling; however, its functional significance cannot be determined from transcript abundance alone. Fatty-acid composition, FADS2 protein abundance, and desaturase activity were not measured. Likewise, the PPAR-β mRNA finding cannot be assumed to reflect corresponding changes in receptor protein abundance or transcriptional activity. Accordingly, the present data do not establish functional alterations in either FADS2- or PPAR-β-related pathways. The contrast between detectable biochemical or histopathological changes and limited gene-expression differences indicates that the selected transcript panel does not fully account for the observed phenotype. Because protein abundance, enzyme activity, post-translational regulation, epigenetic regulation, mitochondrial function, and other metabolic pathways were not assessed, their potential involvement remains speculative and cannot be inferred from the present data.

When considered across biological domains, the present findings are more consistent with a distributed pattern of developmental programming than with disruption of a single metabolic pathway. In male offspring, alterations in glucose regulation and inflammatory markers co-occurred with hepatic histopathological changes, despite relatively limited differences in the selected hepatic lipid-metabolism transcripts. In female offspring, the response pattern was more heterogeneous and involved insulin, lipid-related variables, adipokines, inflammatory markers, and selected hepatic transcripts, with several outcomes also showing maternal diet × propolis interactions. A multidomain pattern of response to maternal cafeteria-diet exposure has also been reported in previous experimental studies. Matuszewska et al. simultaneously demonstrated alterations in offspring adiposity, glucose and insulin profiles, and circulating inflammatory cytokines following maternal cafeteria-diet exposure [17]. Santos et al. integrated metabolic measurements with liver, white-adipose-tissue, and brown-adipose-tissue histology and concluded that the consequences of maternal cafeteria feeding were tissue-specific [15]. Similarly, Vithayathil et al. showed that maternal cafeteria-diet exposure affected fat deposition together with adipokine- and lipid-metabolism-related gene expression in offspring [16], while Akyol et al. demonstrated programming of glucose tolerance in association with maternal cafeteria feeding and reported changes suggestive of altered hepatic insulin-signaling regulation [34]. Taken together with the present findings, these studies are consistent with a pattern of coordinated but heterogeneous alterations across metabolic, inflammatory, adipose, and hepatic domains rather than changes confined to a single pathway. The incomplete concordance among circulating biomarkers, liver histopathology, and hepatic mRNA expression in our study further supports this interpretation. Because male and female offspring were analyzed in separate sex-stratified models, differences in the pattern or statistical significance of findings between the two analyses should not be interpreted as formal evidence of a sex effect. Accordingly, the present findings are described as response patterns observed within each sex rather than as statistically demonstrated sex-dependent effects.

The propolis-related effects were selective rather than universal. In males, the strongest factorial propolis effect was detected for TNF-α, followed by effects on adiponectin and selected organ weights; importantly, the significant glucose interaction was not accompanied by a statistically significant CAFP versus CAF contrast. In females, propolis-related effects involved adipokines, TNF-α, PPAR-β, and FADS2, while several metabolic outcomes showed significant diet × propolis interactions. The within-diet contrasts further refined this interpretation: significant CAFP versus CAF differences were demonstrated for total cholesterol and triglycerides, whereas the corresponding contrasts for insulin and leptin were not significant. Moreover, the leptin interaction was driven by a higher concentration in CONP than CON rather than by a significant difference between CAFP and CAF. Accordingly, the interaction analyses provide evidence for selective, diet-context-dependent biological modulation, but not for generalized metabolic rescue or protection.

The relatively modest and heterogeneous propolis-related effects observed in the present study should also be considered in relation to the selected exposure regimen. The target body-weight-adjusted exposure of 200 mg/kg/day was based on the study by Eraslan et al., who administered propolis to female rats through drinking water at the same body-weight-adjusted exposure [26]. In that study, propolis-containing regimens were associated with significant changes in several serum biochemical outcomes, including glucose, triglycerides, cholesterol, and liver-related enzyme activities. Importantly, however, the authors also reported that the responses were not uniform and that several altered parameters did not return completely toward control values [26]. This previous observation is consistent with the possibility that a 200 mg/kg/day target exposure may produce measurable but endpoint-specific biological effects rather than uniform normalization across metabolic domains. Nevertheless, the previous study involved a toxicological model in non-pregnant rats and therefore cannot establish the optimal exposure for maternal developmental programming. In the present study, no maternal exposure-level comparison or measurement of circulating propolis-derived compounds or metabolites was performed. Consequently, we cannot determine whether the relatively modest offspring responses were related to the selected target exposure, variability in actual exposure through drinking water, bioavailability of the preparation, or the biological characteristics of the maternal programming model. Future studies using multiple exposure levels together with quantified exposure and pharmacokinetic or circulating biomarker measurements are required to clarify these possibilities. Bezerra et al. administered caffeic acid phenethyl ester directly to adult mice with high-fat-diet-induced obesity and observed improved glucose tolerance, hepatic insulin signaling, and steatosis, together with reduced activation of JNK and NF-κB pathways [36]. This study provides mechanistic plausibility for an association between propolis-derived phenolics, inflammation, and insulin signaling. However, it involved direct treatment of obese adult animals with an isolated propolis constituent and therefore cannot be considered direct evidence for the effects of maternal whole-propolis supplementation.

Ikeda et al. showed in cultured 3T3-L1 adipocytes that artepillin C and a related Brazilian propolis compound prevented TNF-α-mediated suppression of adiponectin expression [37]. This cellular finding is relevant to the adiponectin and TNF-α effects observed in the factorial analyses of the present study. Nevertheless, the female CAFP group did not show a straightforward improvement in adiponectin, demonstrating that effects observed in isolated adipocytes cannot be directly extrapolated to a maternal developmental model.

Kanazashi et al. reported that Brazilian propolis supplementation reduced body-fat mass and oxidative-stress markers in community-dwelling older women [38]. Although this trial supports the metabolic bioactivity of propolis in humans, the participants, exposure period, and outcomes differ substantially from those in the present experiment. It therefore provides translational context rather than direct confirmation of a maternal programming effect.

Taken together, these comparisons indicate that anti-inflammatory or adipokine-related mechanisms are biologically plausible, but the present data do not establish a single molecular pathway. The term “partial modulation” is therefore more appropriate than “protection”, “normalization”, or “reversal”.

Importantly, the present study was not designed to establish the molecular mechanisms underlying the observed propolis-related effects. Oxidative-stress markers, antioxidant enzyme activities, intracellular inflammatory signaling pathways, mitochondrial function, and hepatic lipid content were not measured. Therefore, antioxidant, anti-inflammatory, mitochondrial, or lipid-regulatory mechanisms reported in previous studies should be regarded as biologically plausible contextual explanations rather than mechanisms demonstrated by the present data. The current findings identify associations with selected metabolic, inflammatory, histopathological, and transcriptional outcomes but do not establish the molecular pathways responsible for these changes.

The exploratory correlation matrices revealed several coordinated relationships among metabolic, inflammatory, histological, and transcriptional variables that remained statistically significant after FDR correction. In both sexes, glucose was strongly correlated with HOMA-IR. This association is expected in part because glucose is included directly in the HOMA-IR calculation and should therefore not be interpreted as an independent mechanistic finding.

In males, the positive association between TNF-α and hydropic degeneration remained significant after FDR correction, as did the strong relationship between sinusoidal dilatation and congestion. IL-10 was also associated with both congestion and sinusoidal dilatation. These findings indicate coordinated variation among inflammatory and histopathological outcomes but do not establish the direction or mechanism of these relationships. Inflammation is a well-established component of metabolic dysfunction and insulin resistance [39]; however, the present correlations cannot determine whether inflammatory alterations contributed to the histopathological findings or merely co-occurred with them. Similarly, the FDR-significant correlations among PPAR-α, PPAR-β, and SREBP-1c suggest coordinated variation among the measured hepatic transcriptional markers rather than evidence of a specific regulatory pathway.

In females, the strong association between hydropic degeneration and sinusoidal dilatation remained significant after FDR correction, together with correlations between PPAR-α and SREBP-1c and between PPAR-β and SREBP-1c. Insulin was also associated with IL-10 and congestion. Although the use of FDR correction reduces the likelihood that these associations reflect multiple-testing artifacts, the analyses remain exploratory and cross-sectional. Accordingly, these correlations should be regarded as hypothesis-generating and cannot establish causal or mechanistic relationships.

A major strength of the study was the use of one male and one female offspring per litter, with the litter treated as the experimental unit, thereby reducing the risk of pseudoreplication. Standardizing all offspring to chow after weaning allowed the persistence of maternal-programming effects to be assessed without continued cafeteria-diet exposure. The study also integrated biochemical, inflammatory, histological, transcriptional, and organ-morphometric outcomes and reported sex-stratified factorial effect sizes.

Several limitations should be considered. The sample size of eight litters per group limited statistical power for detecting small effects and interactions. In addition, the multi-endpoint design increases the possibility of chance findings when individual outcomes are considered in isolation. Accordingly, the results should be interpreted in relation to the overall pattern of findings and reported effect sizes, and particularly marginal results should be regarded cautiously. Outcomes were assessed at a single adult time point, preventing evaluation of developmental trajectories. Gene expression was assessed only at the mRNA level for five prespecified lipid-metabolism-related genes, and most groupwise comparisons were not statistically significant; therefore, these findings cannot be generalized to broader hepatic transcriptional regulation. Protein abundance, enzyme activity, oxidative-stress markers, antioxidant enzyme activities, intracellular inflammatory signaling pathways, mitochondrial function, hepatic lipid content, and detailed lipidomic outcomes were not assessed. Consequently, the present study cannot establish the molecular mechanisms underlying the observed propolis-related associations. Histopathological scoring was semi-quantitative. The correlation analyses were exploratory; although multiple testing was controlled using the Benjamini–Hochberg FDR procedure separately within each sex, the modest sample size and cross-sectional nature of these analyses limit causal and mechanistic inference. Finally, the results obtained in rats cannot be directly translated into recommendations for propolis supplementation during human pregnancy.

## 5. Conclusions

Maternal cafeteria-diet exposure was associated with persistent metabolic and hepatic alterations in adult offspring despite comparable final body weight. In the male analysis, glucose-regulatory, inflammatory, and histopathological alterations were notable, whereas the female analysis showed a heterogeneous pattern involving lipid, adipokine, and transcriptional outcomes. These observations describe the separate sex-stratified analyses and do not constitute formal evidence of a sex effect.

Maternal propolis supplementation selectively modified several outcomes, including TNF-α, adiponectin, leptin, and selected hepatic gene-expression endpoints, but it did not consistently normalize the cafeteria-associated phenotype. Overall, the findings support selective, endpoint-specific, and context-dependent modulation rather than uniform metabolic or hepatic protection. The hepatic histopathological findings should be interpreted within the limited endpoints assessed and do not establish comprehensive hepatoprotection. The present findings do not establish a specific molecular mechanism underlying these propolis-related effects. Further studies incorporating longitudinal assessments, protein-level analyses, hepatic lipid composition, and mechanistic endpoints are needed before the developmental effects and safety of maternal propolis supplementation can be established.

## Figures and Tables

**Table 1:** Mean daily maternal energy and nutrient intakes across the 14-week intervention period spanning preconception, gestation, and lactation.

Nutrient	Unit	CON	CAF	CAFP	CONP	*p*
Energy intake	kcal/day	55.48 ± 11.14 ^b^	133.20 ± 59.60 ^a^	121.42 ± 47.78 ^a^	59.18 ± 10.45 ^b^	<0.001
Carbohydrate intake	g/day	9.66 ± 1.94 ^b^	17.09 ± 6.07 ^a^	16.06 ± 5.00 ^a^	10.31 ± 1.82 ^b^	<0.001
Protein intake	g/day	2.87 ± 0.58 ^a^	3.06 ± 0.72 ^a^	3.23 ± 0.95 ^a^	3.06 ± 0.54 ^a^	0.798
Fat intake	g/day	0.60 ± 0.12 ^b^	5.59 ± 2.61 ^a^	4.61 ± 1.73 ^a^	0.64 ± 0.11 ^b^	<0.001
Fiber intake	g/day	1.69 ± 0.34 ^a^	1.15 ± 0.19 ^b^	1.40 ± 0.40 ^ab^	1.80 ± 0.32 ^a^	<0.001
Sodium intake	mg/day	54.51 ± 10.94 ^b^	142.98 ± 62.93 ^a^	139.02 ± 64.34 ^a^	58.15 ± 10.27 ^b^	<0.001

Values are presented as mean ± SD (*n* = 8). Groups were compared using one-way analysis of variance (ANOVA), followed by Tukey’s or Games–Howell post hoc test, as appropriate. Within each row, values with different superscript letters are significantly different (*p* < 0.05). CON, control diet; CAF, cafeteria diet; CAFP, cafeteria diet with propolis; CONP, control diet with propolis; SD, standard deviation.

**Table 2:** Relative liver, heart, and kidney weights in dams and male and female offspring.

Cohort	Organ	CON	CAF	CAFP	CONP	Test Statistic	*p*	Effect Size
**Dams**	Liver	4.78 [4.61–5.28] ^ab^	4.47 [4.20–4.60] ^b^	5.11 [5.01–5.62] ^a^	4.25 [4.08–4.76] ^b^	*H*(3) = 10.539	0.015	η*_H_*^2^ = 0.279
	Heart	0.506 [0.461–0.585] ^ab^	0.379 [0.362–0.403] ^b^	0.395 [0.357–0.425] ^b^	0.610 [0.596–0.766] ^a^	*H*(3) = 19.031	<0.001	η*_H_*^2^ = 0.573
	Right kidney	0.505 ± 0.053 ^b^	0.409 ± 0.091^c^	0.496 ± 0.044 ^bc^	0.616 ± 0.074 ^a^	*F*(3, 27) = 11.576	<0.001	η^2^ = 0.563
	Left kidney	0.480 [0.461–0.519] ^b^	0.466 [0.405–0.500] ^b^	0.524 [0.512–0.549] ^ab^	0.609 [0.568–0.678] ^a^	*H*(3) = 16.363	<0.001	η*_H_*^2^ = 0.495
**Male offspring**	Liver	3.72 ± 0.21	3.42 ± 0.61	3.61 ± 0.42	3.64 ± 0.31	*F*(3, 27) = 0.726	0.545	η^2^ = 0.075
	Heart	0.421 [0.382–0.459] ^a^	0.339 [0.333–0.377] ^ab^	0.312 [0.286–0.340] ^b^	0.403 [0.395–0.429] ^a^	*H*(3) = 14.730	0.002	η*_H_*^2^ = 0.434
	Right kidney	0.410 [0.387–0.465] ^ab^	0.350 [0.223–0.393] ^b^	0.398 [0.387–0.410] ^ab^	0.488 [0.455–0.500] ^a^	*H*(3) = 12.339	0.006	η*_H_*^2^ = 0.346
	Left kidney	0.404 [0.364–0.429] ^ab^	0.322 [0.263–0.377] ^b^	0.408 [0.380–0.420] ^ab^	0.431 [0.403–0.472] ^a^	*H*(3) = 9.807	0.020	η*_H_*^2^ = 0.252
**Female offspring**	Liver	3.90 ± 0.32 ^a^	3.55 ± 0.50 ^ab^	3.33 ± 0.35 ^b^	3.81 ± 0.23 ^ab^	*F*(3, 28) = 4.007	0.017	η^2^ = 0.300
	Heart	0.437 ± 0.061 ^ab^	0.382 ± 0.073 ^ab^	0.363 ± 0.053 ^b^	0.462 ± 0.060 ^a^	*F*(3, 28) = 4.428	0.011	η^2^ = 0.322
	Right kidney	0.478 ± 0.073 ^a^	0.422 ± 0.079 ^a^	0.391 ± 0.093 ^a^	0.486 ± 0.026 ^a^	*F*(3, 28) = 3.154	0.040 ^†^	η^2^ = 0.253
	Left kidney	0.442 ± 0.058	0.463 ± 0.075	0.434 ± 0.057	0.421 ± 0.048	*F*(3, 28) = 0.682	0.570	η^2^ = 0.068

Relative organ weight (%) was calculated as organ weight (g)/body weight at euthanasia (g) × 100. Values are presented as mean ± SD for variables analyzed using one-way ANOVA and as median [Q1–Q3] for variables analyzed using the Kruskal–Wallis test. The planned sample size was *n* = 8 per group. For male offspring, relative organ-weight analyses included *n* = 7 in the CAF group because terminal body weight was unavailable for one animal, although all individual organ weights were recorded; all other male and female offspring groups included *n* = 8. Among dams, relative liver, right kidney, and left kidney weight analyses included *n* = 7 in the CONP group because the corresponding organ-weight measurements were unavailable for one animal, whereas the relative heart-weight analysis included *n* = 8 per group. Different superscript letters within a row indicate significant pairwise differences (adjusted *p* < 0.05). One-way ANOVA was followed by Tukey’s HSD test, and the Kruskal–Wallis test was followed by Dunn’s test with Bonferroni adjustment. Effect sizes: η^2^ = SS*_between_*/SS*_total_* for one-way ANOVA; η*_H_*^2^ = (H − k + 1)/(N − k) for the Kruskal–Wallis test. ^†^ Although the omnibus ANOVA for female right kidney weight was significant, no Tukey-adjusted pairwise comparison reached *p* < 0.05. Abbreviations. CON, control diet; CAF, cafeteria diet; CAFP, cafeteria diet supplemented with propolis; CONP, control diet supplemented with propolis; Q1, first quartile; Q3, third quartile.

## Data Availability

The raw data supporting the conclusions of this article are available from the corresponding author upon reasonable request.

## Supplementary Materials

The following supporting information can be downloaded at https://www.mdpi.com/article/10.3390/nu18172881/s1. Table S1: Phytochemical screening of commercial propolis preparations; Table S2: Manufacturer-declared nutritional composition of the standard chow diet (RT-01; DSA Tarım Ürünleri, Türkiye); Table S3: Primer sequences used for quantitative real-time PCR; Table S4: Complete sex-stratified two-way factorial ANOVA results for male offspring; Table S5: Complete sex-stratified two-way factorial ANOVA results for female offspring; Table S6: Within-diet contrasts for outcomes showing significant maternal diet × propolis interactions in the sex-stratified factorial analyses; File S1: Completed ARRIVE 2.0 checklist.
