# Supplementary material for: Beyond Body Weight: Maternal Propolis Selectively Modulates Metabolic and Hepatic Outcomes in Male and Female Rat Offspring Exposed to a Maternal Cafeteria Diet
Source: Nutrients. 2026 Sep 2;18(17):2881. doi: 10.3390/nu18172881 (PMC13567226; doi:10.3390/nu18172881)
Supplement: Supplementary file 1 [file nutrients-18-02881-s001.zip › Supplementary Table S6.pdf]

Supplementary Table S6. Within-diet contrasts for outcomes showing significant maternal diet × propolis interactions in the sex-stratified factorial analyses

Sex	Outcome	Diet stratum	Contrast	Mean difference	95% CI	p value	Hedges' g	95% CI for g
Male	Glucose (mg/dL)	Control	CONP - CON	+16.587	-16.812 to 49.987	0.317	+0.625	-0.340 to 1.569
Male	Glucose (mg/dL)	Cafeteria	CAFP - CAF	-33.457	-68.028 to 1.115	0.057	-0.807	-1.796 to 0.210
Female	Insulin (μU/mL)	Control	CONP - CON	-0.469	-1.033 to 0.096	0.100	-0.731	-1.684 to 0.245
Female	Insulin (μU/mL)	Cafeteria	CAFP - CAF	+0.333	-0.232 to 0.897	0.237	+0.642	-0.325 to 1.587
Female	Total cholesterol (mg/dL)	Control	CONP - CON	+1.993	-1.169 to 5.154	0.207	+0.588	-0.373 to 1.529
Female	Total cholesterol (mg/dL)	Cafeteria	CAFP - CAF	**-3.720**	**-6.882 to -0.558**	**0.023**	**-1.186**	**-2.193 to -0.145**
Female	Triglycerides (mg/dL)†	Control	CONP - CON	+3.959	-10.927 to 18.845	0.590	+0.310	-0.629 to 1.238
Female	Triglycerides (mg/dL)†	Cafeteria	CAFP - CAF	**-22.508**	**-37.394 to -7.621**	**0.004**	**-1.280**	**-2.301 to -0.223**
Female	Leptin (ng/mL)	Control	CONP - CON	**+0.611**	**0.355 to 0.867**	**<0.001**	**+2.069**	**0.849 to 3.244**
Female	Leptin (ng/mL)	Cafeteria	CAFP - CAF	-0.164	-0.420 to 0.092	0.199	-0.718	-1.669 to 0.257

Notes. Mean differences are expressed as the propolis-supplemented condition minus the corresponding non-supplemented condition within each maternal diet stratum (CONP - CON or CAFP - CAF). Pairwise comparisons were obtained from estimated marginal means following significant maternal diet x propolis interactions. Bonferroni adjustment was requested in SPSS; because only two propolis levels were compared within each diet stratum, the adjusted and unadjusted p values were identical. Hedges' g values are reported with 95% confidence intervals; positive values indicate higher values in the propolis-supplemented group and negative values indicate lower values.

† For female triglycerides, the homogeneity-of-variance assumption was violated in the factorial model (Levene's test, $p = 0.002$). Welch unequal-variance tests were therefore performed as sensitivity analyses. The conclusions were unchanged: CONP vs. CON, mean difference = +3.959 mg/dL, 95% CI -9.545 to 17.463, $p = 0.528$; CAFP vs. CAF, mean difference = -22.508 mg/dL, 95% CI -41.653 to -3.362, $p = 0.027$.

Abbreviations. CON, control diet; CONP, control diet with propolis; CAF, cafeteria diet; CAFP, cafeteria diet with propolis; CI, confidence interval.
