# Supplementary material for: Beyond Body Weight: Maternal Propolis Selectively Modulates Metabolic and Hepatic Outcomes in Male and Female Rat Offspring Exposed to a Maternal Cafeteria Diet
Source: Nutrients. 2026 Sep 2;18(17):2881. doi: 10.3390/nu18172881 (PMC13567226; doi:10.3390/nu18172881)
Supplement: Supplementary file 1 [file nutrients-18-02881-s001.zip › Supplementary Table S1.pdf]

Supplementary Table S1. Phytochemical screening of commercial propolis preparations

	**Phenolic Content (mg GAE/g extract) Ethanol-Based Product**	**Phenolic Content (mg GAE/g extract) Water-Based Product**	**Flavonoid Content (mg QE/g extract) Ethanol-Based Product**	**Flavonoid Content (mg QE/g extract) Water-Based Product**
**X**	**44.46**	**18.35**	**19.05**	**15.40**
Y	72.61	17.99	67.95	6.59
Z	43.27	10.67	30.09	6.75
Q	24.83	9.49	16.92	8.34
W	20.96	9.11	14.38	7.54

Values represent the means of duplicate measurements. Total phenolic content was determined using the Folin–Ciocalteu method at 765 nm and expressed as milligrams of gallic acid equivalents per gram of extract (mg GAE/g extract). Total flavonoid content was determined using the aluminum nitrate colorimetric method at 415 nm and expressed as milligrams of quercetin equivalents per gram of extract (mg QE/g extract). Commercial preparations were anonymized using the codes X, Y, Z, Q, and W. Water-based preparation X, which exhibited the highest total phenolic and flavonoid contents among the water-based products, was selected for the animal experiment. GAE, gallic acid equivalents; QE, quercetin equivalents.
