# Supplementary material for: Beyond Body Weight: Maternal Propolis Selectively Modulates Metabolic and Hepatic Outcomes in Male and Female Rat Offspring Exposed to a Maternal Cafeteria Diet
Source: Nutrients. 2026 Sep 2;18(17):2881. doi: 10.3390/nu18172881 (PMC13567226; doi:10.3390/nu18172881)
Supplement: Supplementary file 1 [file nutrients-18-02881-s001.zip › Supplementary Table S2.pdf]

Supplementary Table S2. Manufacturer-declared nutritional composition of the standard chow diet (RT-01; DSA Tarım Ürünleri, Türkiye)

Component	Unit	Declared content
**Energy and macronutrient distribution**		
Energy density	kcal/kg	2646.31
Energy from carbohydrate	% of total energy	69.65
Energy from protein	% of total energy	20.67
Energy from fat	% of total energy	9.68
**Analytical constituents**		
Crude fiber	g/kg	80.5
Ash	g/kg	72.1
Sugars	g/kg	42.2
Starch	g/kg	299.5
Sodium	g/kg	2.6
Calcium	g/kg	10.5
Available phosphorus	g/kg	4.8
Total phosphorus	g/kg	6.8
Potassium	g/kg	8.8
Linoleic acid	g/kg	12.5
**Vitamins and trace nutrients**		
Vitamin A	IU/kg	9600
Vitamin D3	IU/kg	1920
Vitamin E	mg/kg	16
Niacin	mg/kg	24
Choline	mg/kg	240
Iron	mg/kg	20.2
Zinc	mg/kg	47.6
Selenium	mg/kg	0.16
