# Supplementary material for: Beyond Body Weight: Maternal Propolis Selectively Modulates Metabolic and Hepatic Outcomes in Male and Female Rat Offspring Exposed to a Maternal Cafeteria Diet
Source: Nutrients. 2026 Sep 2;18(17):2881. doi: 10.3390/nu18172881 (PMC13567226; doi:10.3390/nu18172881)
Supplement: Supplementary file 1 [file nutrients-18-02881-s001.zip › Supplementary Table S3.pdf]

Supplementary Table S3. Primer sequences used for quantitative real-time PCR

Primer Name	Sequence 5′=> 3′	Tm
CYP7A1-Forward	TCCATCACAATGCCAGTGGTACGACCAG	65
CYP7A1-Reverse	TAGGCACCAGGGTGTGATGGTGGGTATG	
PPAR -α-Forward	TCTGAATGAGCACTTCTAAG	55
PPAR -α-Reverse	CTGTAATTGTCTGAATCCTACTA	
PPAR -β -Forward	TTACTGGAATTCCCATGGTTGACACAGAGA	55
PPAR- β -Reverse	CTGCGTGGATCCTTAGTACATGTCCTTGTA	
SREBP1c -Forward	CAACAACCAAGACAGTGA	58
SREBP1c-Reverse	AGAGAAGCAGGAGAAGAG	
FADS2 -Forward	CCTTCTTCAATGACTGGTT	53
FADS2 -Reverse	CGGCTTCTCTTGGTATTC	
Actin Beta-Forward	CGTTGAACATCCGTAAAGACC	62
Actin Beta-Reverse	GATAGAGCCACCAATCCACA	

CYP7A1: Cholesterol 7 alpha-hydroxylase, PPAR-α: Peroxisome proliferator-activated receptor alpha, PPAR-β: Peroxisome proliferator-activated receptor beta, SREBP1c: Sterol regulatory element-binding protein 1, FADS2: Fatty Acid Desaturase 2
