# Supplementary material for: Beyond Body Weight: Maternal Propolis Selectively Modulates Metabolic and Hepatic Outcomes in Male and Female Rat Offspring Exposed to a Maternal Cafeteria Diet
Source: Nutrients. 2026 Sep 2;18(17):2881. doi: 10.3390/nu18172881 (PMC13567226; doi:10.3390/nu18172881)
Supplement: Supplementary file 1 [file nutrients-18-02881-s001.zip › Supplementary Table S4.pdf]

Supplementary Table S4. Complete sex-stratified two-way factorial ANOVA results for male offspring.

Outcome	Levene p	Propolis main effect			Maternal diet main effect			Diet × propolis interaction		
		**F**	**p**	**ηp^2**	**F**	**p**	**ηp^2**	**F**	**p**	**ηp^2**
Glucose	0.120	0.518	0.478	0.019	5.007	**0.034**	0.156	4.563	**0.042**	0.145
Insulin	0.010	1.985	0.170	0.066	1.540	0.225	0.052	0.815	0.374	0.028
Total cholesterol	0.078	1.119	0.299	0.040	3.014	0.094	0.100	1.285	0.267	0.045
Triglycerides	0.039	0.361	0.553	0.013	1.984	0.170	0.068	0.016	0.900	0.001
Leptin	<0.001	1.244	0.274	0.043	5.353	**0.028**	0.161	0.044	0.835	0.002
Adiponectin	0.316	5.004	**0.033**	0.152	0.003	0.959	0.000	0.008	0.929	0.000
TNF-α	<0.001	11.347	**0.002**	0.288	5.860	**0.022**	0.173	1.032	0.318	0.036
IL-10	0.015	2.241	0.146	0.074	0.550	0.465	0.019	2.654	0.114	0.087
HOMA-IR	0.212	2.911	0.099	0.097	5.779	**0.023**	0.176	2.472	0.128	0.084
CYP7A1 (2^-ΔΔCt)	0.982	0.017	0.898	0.001	2.005	0.168	0.067	2.324	0.139	0.077
PPAR-α (2^-ΔΔCt)	0.010	0.546	0.466	0.019	1.067	0.310	0.037	0.782	0.384	0.027
PPAR-β (2^-ΔΔCt)	0.030	0.053	0.819	0.002	1.834	0.186	0.061	0.488	0.491	0.017
SREBP-1c (2^-ΔΔCt)	0.211	0.019	0.891	0.001	0.096	0.759	0.003	0.323	0.574	0.011
FADS2 (2^-ΔΔCt)	0.044	2.184	0.151	0.072	1.265	0.270	0.043	0.147	0.705	0.005
Relative liver weight	0.722	0.181	0.673	0.006	0.324	0.574	0.011	2.292	0.141	0.076
Relative heart weight	0.124	6.843	**0.014**	0.196	8.938	**0.006**	0.242	1.331	0.258	0.045
Relative right kidney weight	0.033	2.708	0.111	0.088	8.457	**0.007**	0.232	2.157	0.153	0.072
Relative left kidney weight	0.377	4.519	**0.042**	0.139	3.812	0.061	0.120	2.946	0.097	0.095
Final body weight	0.118	1.439	0.241	0.051	0.807	0.377	0.029	0.246	0.624	0.009

Notes: Two-way ANOVA assessed the main effects of maternal diet (control vs. cafeteria diet) and propolis supplementation (absent vs. present), and their interaction. Levene's test was used to assess homogeneity of variance. ηp^2, partial eta squared. Gene-expression outcomes are expressed as 2^-ΔΔCt fold-change values. Statistically significant factorial effects ($p < 0.05$) are shown in bold; Levene p values are provided for assumption checking.
