# Supplementary material for: Beyond Body Weight: Maternal Propolis Selectively Modulates Metabolic and Hepatic Outcomes in Male and Female Rat Offspring Exposed to a Maternal Cafeteria Diet
Source: Nutrients. 2026 Sep 2;18(17):2881. doi: 10.3390/nu18172881 (PMC13567226; doi:10.3390/nu18172881)
Supplement: Supplementary file 1 [file nutrients-18-02881-s001.zip › Supplementary Table S5.pdf]

Supplementary Table S5. Complete sex-stratified two-way factorial ANOVA results for female offspring.

Outcome	Levene p	Propolis main effect			Maternal diet main effect			Diet × propolis interaction		
		**F**	**p**	**ηp²**	**F**	**p**	**ηp²**	**F**	**p**	**ηp²**
Glucose	0.537	0.146	0.705	0.005	3.161	0.086	0.101	0.563	0.459	0.020
Insulin	0.691	0.122	0.730	0.004	5.741	**0.023**	0.170	4.228	**0.049**	0.131
Total cholesterol	0.828	0.626	0.435	0.022	2.505	0.125	0.082	6.848	**0.014**	0.197
Triglycerides	0.002	3.257	0.082	0.104	2.530	0.123	0.083	6.632	**0.016**	0.191
Leptin	0.155	6.387	**0.017**	0.186	18.151	**<0.001**	0.393	19.259	**<0.001**	0.408
Adiponectin	0.104	8.676	**0.006**	0.237	0.310	0.582	0.011	1.076	0.308	0.037
TNF-α	0.147	7.267	**0.012**	0.206	1.245	0.274	0.043	2.876	0.101	0.093
IL-10	0.234	1.885	0.181	0.063	5.337	**0.028**	0.160	2.425	0.131	0.080
HOMA-IR	0.498	0.073	0.789	0.003	0.283	0.599	0.010	0.231	0.635	0.008
CYP7A1 (2^-ΔΔCt)	0.003	0.927	0.344	0.032	1.419	0.244	0.048	0.081	0.779	0.003
PPAR-α (2^-ΔΔCt)	0.292	2.473	0.127	0.081	3.993	0.055	0.125	0.303	0.586	0.011
PPAR-β (2^-ΔΔCt)	0.186	4.342	**0.046**	0.134	0.048	0.827	0.002	0.909	0.349	0.031
SREBP-1c (2^-ΔΔCt)	0.033	0.471	0.498	0.017	0.364	0.551	0.013	0.714	0.405	0.025
FADS2 (2^-ΔΔCt)	0.018	7.787	**0.009**	0.218	0.036	0.851	0.001	2.120	0.157	0.070
Relative liver weight	0.105	2.055	0.163	0.068	8.202	**0.008**	0.227	0.264	0.611	0.009
Relative heart weight	0.453	0.122	0.729	0.004	7.978	**0.009**	0.222	0.022	0.882	0.001
Relative right kidney weight	0.205	0.899	0.351	0.031	12.529	**0.001**	0.309	0.000	0.991	0.000
Relative left kidney weight	0.942	2.339	0.137	0.077	0.003	0.954	0.000	0.750	0.394	0.026
Final body weight	0.001	0.465	0.501	0.016	0.483	0.493	0.017	1.365	0.253	0.046

Notes: Two-way ANOVA assessed the main effects of maternal diet (control vs. cafeteria diet) and propolis supplementation (absent vs. present), and their interaction. Levene's test was used to assess homogeneity of variance. ηp², partial eta squared. Gene-expression outcomes are expressed as 2^-ΔΔCt fold-change values. Statistically significant factorial effects ($p < 0.05$) are shown in bold; Levene p values are provided for assumption checking.
